# Neo-functionalization of a *Teosinte branched 1* homologue mediates adaptations of upland rice

**DOI:** 10.1038/s41467-019-14264-1

**Published:** 2020-02-05

**Authors:** Jun Lyu, Liyu Huang, Shilai Zhang, Yesheng Zhang, Weiming He, Peng Zeng, Yan Zeng, Guangfu Huang, Jing Zhang, Min Ning, Yachong Bao, Shilei Zhao, Qi Fu, Len J. Wade, Hua Chen, Wen Wang, Fengyi Hu

**Affiliations:** 1 State Key laboratory for Conservation and Utilization of Bio-Resources in Yunnan, Research Center for Perennial Rice Engineering and Technology of Yunnan, School of Agriculture, Yunnan University, 650091 Kunming, Yunnan China; 2 State Key Laboratory of Genetic Resources and Evolution, Kunming Institute of Zoology, Chinese Academy of Sciences, 650223 Kunming, Yunnan China; 3 State Key Laboratory of Quality Research in Chinese Medicine, Institute of Chinese Medical Sciences, University of Macau, Macau, China; 4 Beijing Institute of Genomics, Chinese Academy of Sciences, Beijing, China; 5 The University of Queensland, School of Agriculture and Food Sciences, Brisbane, QLD 4072 Australia; 6 Center for Excellence in Animal Evolution and Genetics, Chinese Academy of Sciences, 650223 Kunming, Yunnan China; 7 Center for Ecological and Environmental Sciences, Key Laboratory for Space Bioscience & Biotechnology, Northwestern Polytechnical University, 710072 Xi’an, China

**Keywords:** Agricultural genetics, Evolutionary developmental biology, Plant breeding, Plant evolution

## Abstract

The rice orthologue of maize domestication gene *Teosinte branched 1* (*Tb1*) affects tillering. But, unlike maize *Tb1* gene, it was not selected during domestication. Here, we report that an *OsTb1* duplicate gene (*OsTb2*) has been artificially selected during upland rice adaptation and that natural variation in *OsTb2* is associated with tiller number. Interestingly, transgenic rice overexpressing this gene shows increased rather than decreased tillering, suggesting that *OsTb2* gains a regulatory effect opposite to that of *OsTb1* following duplication. Functional analyses suggest that the OsTb2 protein positively regulates tillering by interacting with the homologous OsTb1 protein and counteracts the inhibitory effect of OsTb1 on tillering. We further characterize two functional variations within *OsTb2* that regulate protein function and gene expression, respectively. These results not only present an example of neo-functionalization that generates an opposite function following duplication but also suggest that the *Tb1* homologue has been selected in upland rice.

## Abstract (web summary)

A transposon insertion in the regulatory region of maize *Tb1* gene leads to increased apical dominance and a reduction of tillering. Here, the authors showed that a duplicated rice *Tb1* orthologue, *OsTb2*, has gained a regulatory effect on tillering opposite that of *OsTb1* during artificial selection only in upland japonica rice.

## Introduction

Modern civilization is built on a foundation of domesticated crops and animals that have been the main source of calories for humans for more than 10,000 years. Multiple domesticated crops often share similar domestication traits compared to their wild relatives, such as loss of seed shattering and dormancy, increased fruit size, or alterations of plant architecture; this phenomenon is referred to as domestication syndrome. An increase in apical dominance is an important example of domestication syndrome that occurs in many gramineous crops. Domesticated maize, sorghum, rice, wheat, and foxtail millet all show an increase in apical dominance and a corresponding reduction in shoot branching compared to their wild counterparts^1^. The well-established domestication gene *Teosinte branched 1* (*Tb1*) was originally found to result in increased apical dominance in maize^2^. QTLs containing *Tb1* orthologous loci in sorghum, foxtail millet, wheat and pearl millet were later discovered to account for tiller variation under domestication^3–5^. However, these studies based on QTL analysis do not unequivocally demonstrate whether the underlying causal gene is a *Tb1* orthologue or not. In rice, the *Tb1* orthologous gene *OsTb1*, located on chromosome 3, was shown by mutant analysis to impact tiller branching but was suggested to not be related to rice domestication^6,7^ because this locus was not selected during domestication. A previous whole-genome scan for domestication genes in rice conducted by large-scale genome resequencing also detected no signals for artificial selection around this region^8,9^. Although it has been realized that the increase in apical dominance constitutes parallel morphological evolution in cereal crops, it remains elusive whether this parallel domestication has a similar genetic basis.

There are two rice subspecies *Oryza sativa japonica* and *indica* that exhibit different tillering abilities, with the *japonica* subspecies tending to have fewer tillers than *indica*. Rice also has two ecotypes, upland and irrigated ecotypes, which are adapted to rainfed upland conditions and well-watered conditions, respectively. Our previous analysis of upland rice genomes and irrigated rice genomes revealed that another gene, which is a paralogue of *OsTb1* located on rice chromosome 9 (hereafter referred to as *OsTb2*), is highly differentiated between the two ecotypes^10^. Upland rice varieties are generally *japonica* and tend to exhibit taller plant architecture, better-developed roots and fewer tillers compared to their irrigated counterpart (Supplementary Fig. 1). It has been known that in upland rice fewer tillers is an adaptive architecture because upland varieties with a small number of tillers tend to have longer deep roots and larger panicles than those with profuse tillers^11,12^. Given that the two ecotypes have apparently different tillering abilities^10^, it is tempting to speculate that *OsTb2* might regulate rice tillering.

Gene duplication is a major way whereby new genes originate. *OsTb2* and *OsTb1* are highly homologous and appear to be the two most closely related gene copies in the rice genome that likely diverged from a gene duplication event. After duplication, the new gene copy will be functionally redundant with the old copy in the short term, while in the long term, it can become a pseudogene or may be lost^13^. Alternatively, in some scenarios, the new copy obtains a new function during evolution, a process referred to as neo-functionalization^14,15^. It is important to test whether *OsTb2* has retained the same function as *OsTb1* in repressing tillering. *DWARF14* (*D14*) is a gene involved in strigolactone signalling and negatively regulates rice tillering^16^. Previous studies showed that *OsTb1* represses tillering by interacting with *OsMADS57* to promote *D14* expression^17^. Whether *OsTb2* plays a similar role in regulating rice tillering remains to be elucidated.

In this study, we present evidence that *OsTb2* has evolved a function opposite to that of its paralogue *OsTb1*. Unlike *OsTb1*, which is a tillering inhibitor, *OsTb2* is a positive regulator of tillering. We show that OsTb2 likely functions by interfering with the inhibitory effect of OsTb1 on tillering. Moreover, we find that a 3 base pair (bp) indel in the coding region of *OsTb2* is divergent between the *japonica* and *indica* subspecies and that in *japonica*, the 3 bp insertion enhances the function of OsTb2 in promoting tillering. More interestingly, another functional variation is a T to C mutation that has been selected and fixed in upland rice. By reducing the expression of *OsTb2*, this derived C allele has likely contributed to the dryland adaptation of upland rice by reducing tillers and increasing grain yield per panicle, generating an upland-adaptive plant architecture that was favoured and selected by humans. Our findings not only provide another vivid example of gene neo-functionalization but also demonstrate that paralogous genes with opposite functions might be selected during domestication and breeding.

## Results

### *OsTb2* is differentiated between upland and irrigated rice

**Fig. 1:**
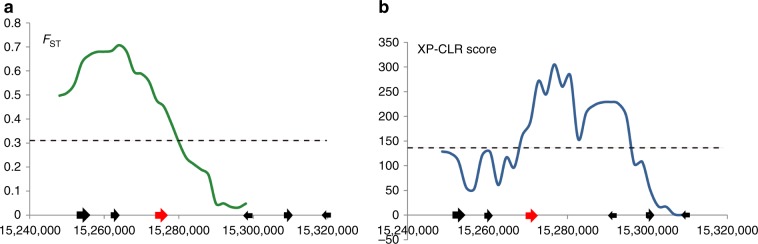
Artificial selection signal around the *OsTb2* region. Peak signals (red arrows) were found by *F*_ST_ (**a**) and XP-CLR (**b**) assessment, respectively. The artificial selection signals were detected based on the *F*_ST_ value (**a**) and the cross-population composite likelihood ratio test (XP-CLR, **b**). The *F*_ST_ value and XP-CLR score were calculated window by window (see Methods), and we then choose the windows with genome-wide top 5‰ values as candidate regions with selection signals. The dotted horizontal lines indicate the threshold of genome-wide top 5‰ value.

Upland and irrigated rice ecotypes display significant differentiation in tillering ability. In our previous comparative genomic study^10^, we found that *Os09g0410500* on chromosome 9, a homologue of the maize *Tb1* gene, was highly differentiated between the two ecotypes. The rice orthologue of the maize *Tb1* gene, *OsTb1*, is located on chromosome 3, showing the highest homology to maize *Tb1* among the rice genes (See Methods). We therefore referred to the *Tb1* homologue on chromosome 9 as *OsTb2*. *F*_ST_ and XP-CLR were used to assess the artificial selection signature around *OsTb2* (40 kb upstream to 40 kb downstream) (see Methods). Both *F*_ST_ and XP-CLR displayed a peak signal around *OsTb2*, and the empirical *P*-values of both tests are below 5‰ (Fig. 1), indicating *OsTb2* was probably selected during the differentiation of upland *japonica* and irrigated *japonica* rice.

Since high population differentiation of a gene region can be caused by multiple other factors such as allele surfing, hierarchical population structure etc.^18^, we further checked the allele frequency spectra of SNPs from the vicinity of the putative causal mutations (see the next section for details about the putative causal mutations). We observed a U-shape pattern of the derived allele frequency spectra (AFS) in upland *japonica*, and the pattern decayed with the increasing distance from the causal mutations (Supplementary Fig. 2), which is a signal of the hitch-hiking effect^19,20^. We further performed a nonparametric Kolmogorov-Smirnov test to show that with the increasing distance from the focal mutation, the U-shape AFS pattern of SNPs in the sliding windows also decays and become similar to the background AFS pattern (Supplementary Fig. 3), supporting the hitch-hiking event. Moreover, we used the Hudson-Kreitman-Aguadé (HKA) test to screen for genome-wide recently selected genes (see Methods), and *OsTb2* was found to be among the 301 selected genes (HKA test *P*-value = 0.019). Performing genome scan using the SweeD program also uncovered a significant likelihood value (ranking top 1.6%) in upland *japonica*, but an insignificant likelihood value (ranking top 17.3%) in irrigated *japonica* (Supplementary Fig. 4). These multiple lines of evidences strongly support that *OsTb2* was under selection during the cultivation of upland *japonica* rice. As shown by our previous phylogenetic analysis^10^, upland *japonica* evolved from irrigated *japonica*. The artificial selection signature therefore suggested that *OsTb2* might have been selected during evolution from irrigated rice to upland rice. Considering that the *OsTb1* homologues, found in maize and other crops, have been reported to account for the change in apical dominance and that upland rice accessions actually have a significantly lower tillering ability than irrigated accessions^10^, we were interested in determining whether *OsTb2* also impacts tiller number in rice and whether it was selected during the improvement of upland rice.

### *OsTb2* is associated with rice tillering ability

**Fig. 2:**
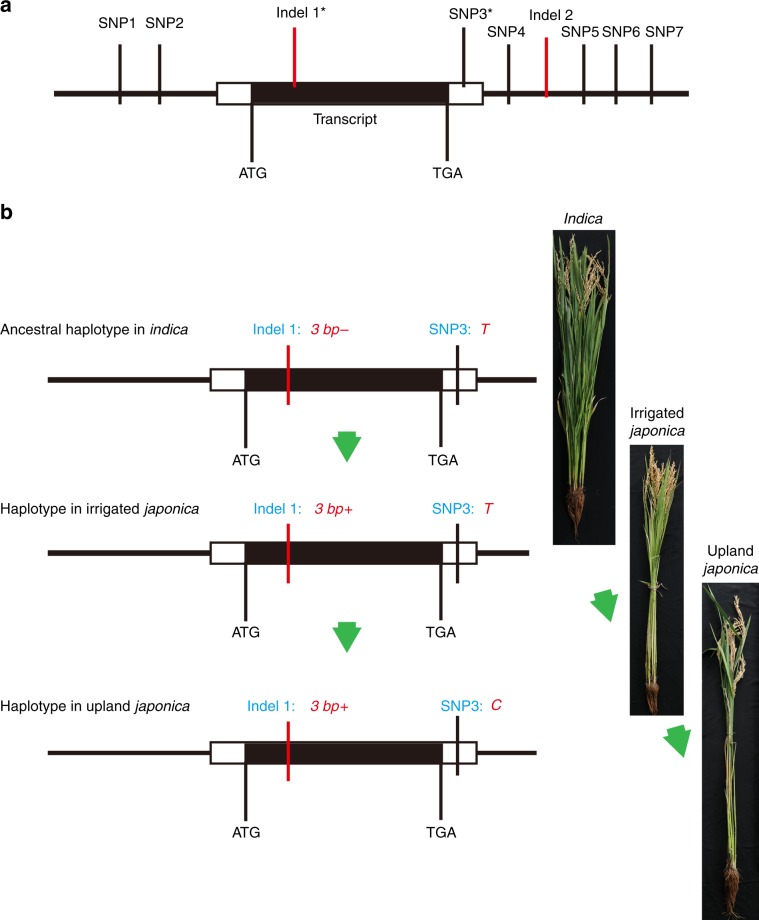
Variants and haplotypes of *OsTb2*. **a** Structure and polymorphic sites of *OsTb2*. Two indels and seven SNPs were found in the *OsTb2* gene. Indel I and SNP3 (bold with asterisk) are both significantly associated with tiller numbers. **b** Indel I and/or SNP3 in the *OsTb2* gene may be functional variants for which three haplotypes were observed in the germplasm. The 3bp-/T haplotype is present in *indica* and wild rice, and thus likely to be the ancestral haplotype. The 3bp+/T haplotype is mainly found in irrigated *japonica*, and the 3bp+/C haplotype is specific to upland *japonica*, consistent with the evolutionary viewpoint that upland *japonica* evolved from irrigated *japonica*.

To identify the polymorphic sites of *OsTb2*, we sequenced this gene in 84 upland and 82 irrigated accessions (Supplementary Data 1) using Sanger sequencing. In total, seven SNPs and two indels were identified (Fig. 2a). To investigate the association between *OsTb2* and tillering ability, we grew 132 of the above sequenced accessions and collected phenotypic data on their tiller numbers at 40 and 50 days after germination (DAG). We then tested the association between the SNPs/indels and tiller number phenotypes. As shown in Table 1, among the nine polymorphic sites, only Indel I and SNP3 were significantly associated with tiller number at both 40 DAG and 50 DAG. Moreover, these associations presented the smallest *P-*values among all the variants, indicating that Indel I and/or SNP3 is likely to be the functional variant(s) (Table 1). The two variants produce three haplotypes (Fig. 2b). Considering that hitch-hiking variants tightly linked with causal variants also have the potential to be associated with phenotypes, it remains to be determined whether only one or both variants are functional.

**Table 1:** Association between *OsTb2* polymorphic sites and tiller numbers.

Polymorphic sites	Position on chr09	Segregating genotypes	*P*-value^a^ (40 DAG)	*P*-value (50 DAG)
SNP1	15272303	C/T	0.08713	0.5717
SNP2	15272752	G/A	0.07238	0.8106
indel1	15273436~15273438	3bp+/3bp−^b^	0.01099	1.30E-11
SNP3	15274099	T/C	3.80E-05	2.55E-12
SNP4	15274295	T/C	0.4748	0.0003731
indel2	15274601~15274603	3bp+/3bp-	0.3998	5.88E-08
SNP5	15274707	G/A	0.2722	1.38E-08
SNP6	15274798	C/T	rare SNPs	rare SNPs
SNP7	15274921	G/A	0.2306	9.45E-07

Source data are provided as a Source Data file.

^a^For each polymorphic site, we divided the accessions into two homozygous groups. Students’ *t*-test was then used to assess the differences in tiller numbers and determine significant *P* values between two groups. Indel I and SNP3 are significantly associated with tiller numbers at both 40 DAG and 50 DAG. The effect sizes are shown in Supplementary Table 1.

^b^3bp+ refers to the three base pair insertion; 3bp− refers to the three base pair deletion. The positions on Chr09 are in reference to genome version IRGSP 5.0.

Association analysis can sometimes yield a false-positive result due to population structure^21^. Thus, we further tested the association between *OsTb2* alleles and tiller number in segregating populations. We used an F_8_ recombinant inbred line (RIL) population obtained by crossing the upland variety IRAT104 and the irrigated variety IR64, which segregate for both the Indel I and SNP3 markers. We genotyped the 134 lines in the F_8_ RIL populations using derived cleaved amplified polymorphic sequence (dCAPS) markers^22^ (see Methods) and grew those lines in irrigated and upland conditions to observe their phenotypes. Severely distorted segregation phenomena were observed for both Indel I and SNP3 loci. For the Indel I locus, 32 lines had a 3bp+ (3 bp insertion) genotype, while 96 lines had a 3bp− (3 bp deletion) genotype (the other six lines were heterozygous). When tiller numbers were compared between the Indel I-3bp+ lines and Indel I-3bp− lines, we observed a significant increase in tillers for the Indel I-3bp+ lines compared to lines with the Indel I-3bp− genotype (Student’s *t*-test, *P* *=* 0.039; Table 2). For the SNP3 site, the RIL-F_8_ population was so skewed towards SNP3-T that we found only four lines with SNP3-C, making it difficult to statistically test its association with tiller number. However, we found one individual, RIL116, that was heterozygous for both Indel I and SNP3. Therefore, we selfed this individual to produce a near-isogenic F_2_ population segregating for both Indel I and SNP3.

**Table 2:** Conditional analysis for Indel I and SNP3 in natural and F_8_-RIL populations.

Analysis	Natural population		F_8_-RIL population	
	Comparison of Indel I-3bp+ with Indel I-3bp− conditioning on SNP3-T	Comparison of SNP3-T with SNP3-C conditioning on Indel I-3bp+	Comparison of Indel I-3bp+ with Indel I-3bp-	Comparison of Indel I-3bp+ with Indel I-3bp− conditioning on SNP3-T
*P*-value (40 DAG)	0.6954	0.0069	0.1343	0.1509
Mean value difference	4.17%	29.50%	12.70%	14.50%
Variance	9.1	8.4	9.4	9.9
Effect size	0.17	3.22	0.62	0.72
*P*-value (50 DAG)	1.60E-05	0.0003	3.90E-02	0.2032
Mean value difference	30.70%	32.10%	11.20%	6.50%
Variance	81.3	44.9	43.9	40.2
Effect size	5.02	3.47	1.41	0.78
Tiller number comparison	Indel I-3bp+ <^a^ Indel I-3bp−	SNP3-C < SNP3-T	Indel I-3bp+ >^b^ Indel I-3bp−	Indel I-3bp+ > Indel I-3bp-

Source data are provided as a Source Data file.

^a^Less than.

^b^More than.

The derived near-isogenic F_2_ population included 451 individuals, all of which were grown to be phenotyped and genotyped. In this near-isogenic F_2_ population, distorted segregation was also observed for SNP3. Among the 451 individuals, we identified 135 individuals with the SNP3-T genotype, but only 60 individuals with the SNP3-C genotype, and the rest were heterozygous. For Indel I, 60 homologous 3bp− individuals and 75 homologous 3bp+ individuals were genotyped. All of these individuals were phenotyped twice (40 and 50 DAG). A conditional association study was then conducted to examine the association between SNP3/Indel I and tiller number. The results showed that the SNP3-C genotype had significantly fewer tillers than did the SNP3-T genotype under the Indel I-3bp+ condition (Student’s *t*-test, *P* = 6.15E-05 at 50 DAG; Table 3), while Indel I-3bp+ plants had significantly more tillers than Indel I-3bp− plants (Student’s *t*-test, *P* = 0.0193 at 50 DAG; Table 3). This result further confirmed that the derived Indel I-3bp + allele in *japonica* corresponded to an increase in tiller number, while the derived SNP3-C allele in upland *japonica* was associated with a reduced number of tillers. The findings that at 40 DAG, the SNP3 locus, but not the Indel I locus, was marginally significantly associated with tiller number (Student’s *t*-test, *P* = 0.1191; Table 3) and that the Indel I locus became significant only at 50 DAG suggested that, consistent with what we observed in the natural population association study, SNP3 might exhibit a function around the early tillering stage (40 DAG) and that Indel I probably affects tillering around the late tillering stage (50 DAG).

**Table 3:** Conditional analysis for Indel I and SNP3 in a near-isogenic F_2_ population.

Analysis	Comparison of Indel I-3bp+ with Indel I-3bp- conditioning on SNP3-T	Comparison of SNP3-T with SNP3-C conditioning on Indel I-3bp+
*P*-value (40 DAG)	0.3724	1.19E-01
Mean value difference	7.59%	12.90%
Variance	19.5	13.1
Effect size	0.47	0.82
*P*-value (50 DAG)	0.02	6.15E-05
Mean value difference	15.40%	22.80%
Variance	70.9	45.1
Effect size	1.64	2.77
Tiller number comparison	Indel I-3bp+ >^a^ Indel I-3bp−	SNP3-C <^b^ SNP3-T

Source data are provided as a Source Data file.

^a^More than.

^b^Less than.

### *OsTb2*^*3bp+*^ can increase rice tiller number

**Fig. 3:**
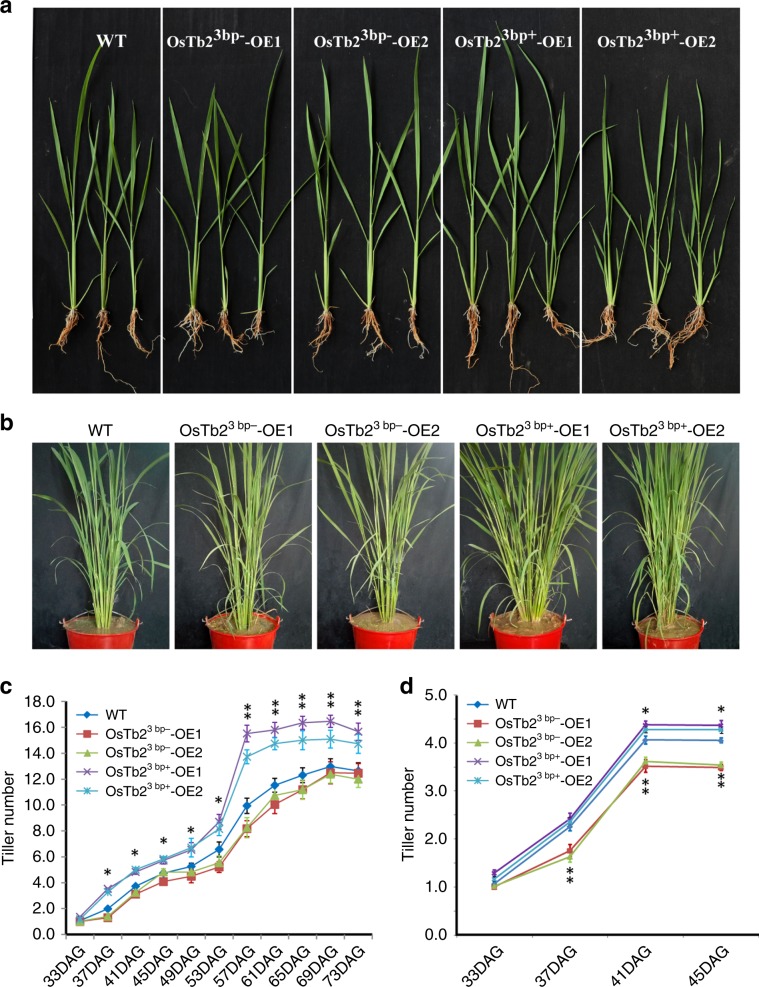
*OsTb2*^*3bp+*^ can positively regulate rice tillering. **a** Phenotypes of transgenic plants overexpressing two genotypes (3 bp+ and 3bp-) of *OsTb2* at 30 DAG under irrigated conditions. WT, wild type. **b** Phenotypes of transgenic plants overexpressing two genotypes of *OsTb2* at 65 DAG under irrigated conditions. WT, wild type. **c** Tiller number of transgenic plants overexpressing both *OsTb2*^*3bp+*^ and *OsTb2*^*3bp−*^ at different DAG under irrigated conditions. **d** Tiller numbers of transgenic plants overexpressing both *OsTb2*^*3bp+*^ and *OsTb2*^*3bp−*^ at different DAT under dryland conditions. Each value in c, d represents the mean ± s.d. (n = 50 plants). Student’s *t*-test analysis indicated a significant difference (compared with the WT control, *P < 0.05, **P < 0.01).

We examined *OsTb2* expression patterns in different tissues at 40 DAG and 50 DAG stages using qRT-PCR. The results showed that *OsTb2* was predominantly expressed in the basal tiller node with a relatively lower expression in leaf blade and sheath. Higher expression of *OsTb2* in the basal tiller node at 40 DAG than 50 DAG indicated that *OsTb2* starts to function from the early stage of tillering (Supplementary Fig. 5). To validate the function of *OsTb2*, we cloned the gene sequences of IRAT104 (*OsTb2*^*3bp+*^) and IR64 (*OsTb2*^*3bp−*^) into the overexpression vector pCUBI-1390, driven by the *Ubiquitin* promoter, which was then transformed into Nipponbare. Multiple positive transgenic lines were obtained by hygromycin B screening. Gene expression was greatly increased in the *OsTb2*^*3bp+*^*-OE1~6* and *OsTb2*^*3bp−*^*-OE1~6* lines compared to the control lines, as shown in Supplementary Fig. 6a. Two transgenic lines for each genotype as well as control lines (both negative lines and WT) were then planted in dryland and irrigated environments in two growth seasons for phenotypic examination. Accordingly, it was found that the transgenic lines overexpressing *OsTb2*^*3bp+*^ had significantly more tillers than the control lines (Fig. 3 and Supplementary Fig. 6b, c), supporting the hypothesis that unlike its homologue *Tb1*, which is a tiller suppressor, *OsTb2* is a tiller enhancer. The transgenic lines overexpressing *OsTb2*^*3bp−*^ had slightly fewer (but not significantly) tillers, implying that the *OsTb2*^*3bp−*^ genotype had a limited effect on tiller number.

Irrigated *japonica* often has fewer tillers than irrigated *indica* rice likely due to their different genetic composition. Our transgenic experiments showed that the *japonica*-specific *OsTb2*^*3bp+*^ could partially increase tiller number (Fig. 3b, d) and thus could alleviate tiller suppression by other genes in the *japonica* background. This allele might have been fixed in *japonica* because it could alleviate tiller suppression or due to random genetic drift.

### Indel I alters OsTb2’s regulatory effects on *D14* expression

We then wondered how the Indel I mutation might alter the function of *OsTb2* in affecting tiller number. Considering that Indel I-3bp+ causes a single amino acid insertion in the TCP binding domain of this *OsTb2* transcription factor and that in silico prediction hinted that this insertion might have changed the peptide secondary structure (Supplementary Fig. 7), we hypothesized that I-3bp+ may have altered the function of OsTb2 by altering its structure.

**Fig. 4:**
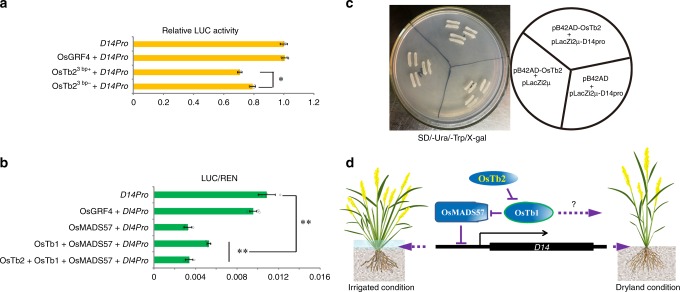
OsTb2^3bp+^ negatively regulates *D14* via the OsTb1-OsMADS57-D14 pathway. **a** Effects of OsTb2^3bp+^ and OsTb2^3bp*−*^ on the transcriptional regulation of *D14* in rice protoplasts. **b** Effects of OsTb2^3bp+^ on the transcriptional regulation of *D14* via the OsTb1-OsMADS57-D14 pathway in *Nicotiana benthamiana*. OsGRF4 is used as a negative control for detecting the effects of OsTb2 on *D14pro::LUC* activity. Each relative luciferase activity of *D14pro::LUC* value in a and b represents the mean ± s.d. (n = 10 biologically independent samples). Student’s *t*-test analysis indicated a significant difference (*P < 0.05, **P < 0.01). **c** In the yeast one-hybrid assay, OsTb2 fusion proteins fail to activate *D14pro*::*LacZ* reporter gene expression in yeast. The right diagrammatic drawing indicates the yeast strains transformed with related plasmids. **d** A schematic model depicts that OsTb2 interacts with OsTb1 to regulate the expression of *D14*. OsMADS57 directly represses the expression of *D14*; OsTb2 interacts with OsTb1 to alleviate OsTb1’s inhibition on OsMADS57, which consequently reduces the expression of *D14* and increases tillers.

As our data showed that *OsTb2*^***3bp+***^ had a function (i.e., promoting tillering) antagonistic to the *Tb1* orthologue *OsTb1*, we next asked whether *OsTb2* influenced gene expression in an opposite manner. To determine whether the 3 bp insertion affected the function of the *OsTb2* transcription factor, we carried out a transient expression assay using a luciferase reporter system. *D14* expression could promote apical dominance and reduce tillers. We found that the extent of D14::LUC expression was reduced by cotransformation with *OsTb2* (Fig. 4a). The transient expression assays showed that both *OsTb2*^*3bp+*^ and *OsTb2*^*3bp−*^ indeed impacted the expression of *D14*, and *OsTb2*^*3bp+*^ exerted a significantly greater inhibitory effect than *OsTb2*^*3bp−*^ (Fig. 4a). It was previously shown that OsTb1 represses tillering by increasing the expression of *D14*. Therefore, it is likely that OsTb2 represses *D14* by counteracting the positive regulation of *D14* transcription by OsTb1. The yeast one-hybrid (Y1H) assay verified that OsTb2 could not bind to *D14* promoter directly (Fig. 4c), which implied that OsTb2 reduced expression in other ways. To determine how OsTb2 repressed *D14* expression, transient expression assays were further used to test whether OsTb2 plays a role in repressing *D14* via the OsTb1-OsMADS57 pathway. When OsTb2 was coexpressed with OsTb1 and OsMADS57, the expression of the cotransformed reporter gene *D14pro::LUC* indicated that OsTb2 may neutralize the inhibition of OsTb1 on OsMADS57, which directly binds the *D14* promoter to inhibit its transcription (Fig. 4b, d). The results also showed that the two Indel I genotypes resulted in significant differences in *D14* expression (Student’s *t*-test, *P*_*OsTb2*_ = 0.0218, Fig. 4a): the 3bp+ genotype corresponded to a lower level of *D14*, which was consistent with the 3bp+ genotype yielding more tillers. Therefore, we concluded that *OsTb2* reduces the expression of *D14*, which then consequently increase tiller number. The 3 bp insertion that occurred in *japonica* promoted the repression of *D14* by OsTb2, thus representing a genotype yielding an increased tiller number.

### OsTb2 binds to OsTb1 and offset OsTb1’s tiller suppression

**Fig. 5:**
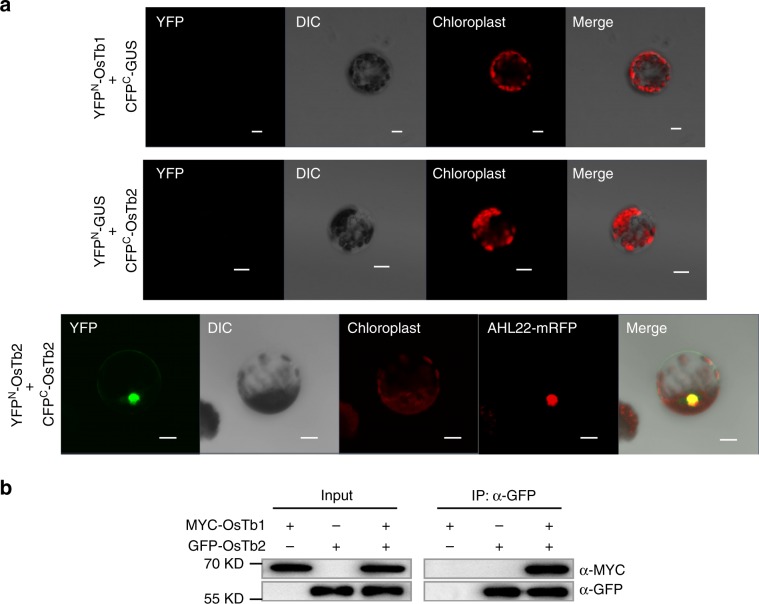
OsTb2 interacts with OsTb1 in the nucleus. **a** YFP^N^-OsTb1 and CFP^C^-OsTb2 were expressed in a pairwise manner in rice protoplasts and exhibited a direct interaction in the nucleus, in which AHL22-mRFP was used as the nuclear marker protein; CFP^C^-GUS and YFP^N^-GUS fusion proteins were used as negative controls and were coexpressed with YFP^N^-OsTb1 and CFP^C^-OsTb2, respectively, in rice protoplasts. DIC indicates differential interference contrast transmission; the merged image is also shown; scale bar, 20 µm. **b** Coimmunoprecipitation assays of OsTb1 and OsTb2. Protein extracts from rice protoplasts harbouring MYC-OsTb1 and GFP-OsTb2 were coimmunoprecipitated by anti-GFP beads and detected by anti-GFP and anti-MYC antibodies. Protoplasts transformed with single MYC-OsTb1 or GFP-OsTb2 were used as a negative control. The source data underlying Fig. 5b are provided as a Source Data file.

TCP genes encode plant-specific transcription factors with a bHLH motif that allows DNA binding and protein–protein interactions, forming homodimers or heterodimers^23,24^. Therefore, we asked whether OsTb1 interacts with OsTb2 in planta. BiFC assays indicated that the interaction between OsTb2 and OsTb1 occurred in the *Nicotiana benthamiana* nucleus (Fig. 5), which was consistent with the nuclear subcellular localization of OsTb2 (Supplementary Fig. 8). Therefore, OsTb2 was able to form a heterodimer with OsTb1. CoIP tests revealed that the protein complexes pulled down using anti-α-GFP agarose were recognized by an anti-α-MYC antibody in lines cotransformed with GFP-OsTb2 and MYC-OsTb1 (Fig. 5); i.e., OsTb2 could bind to OsTb1 in planta. It was previously found that OsTb1 represses tillering by increasing the expression of *D14*^17^. Therefore, OsTb2 probably represses *D14* expression by counteracting the positive regulatory effect of OsTb1 on *D14*, ultimately increasing tiller number.

### SNP3 C-allele confers reduced *OsTb2* expression and tillers

**Fig. 6:**
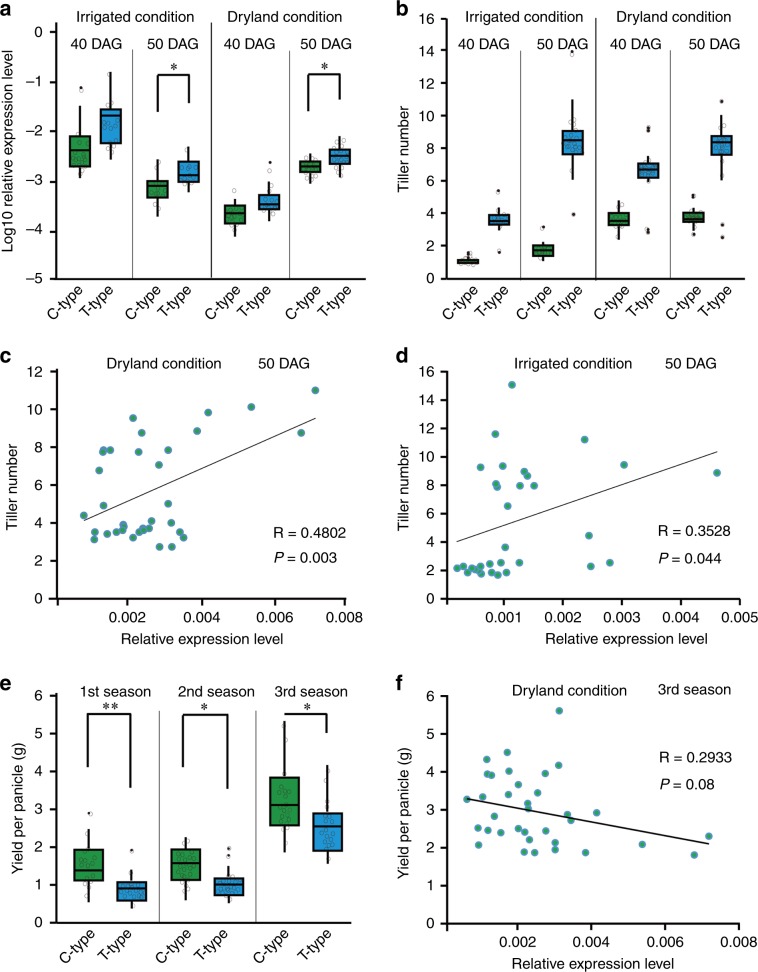
SNP3 is associated with *OsTb2* gene expression and phenotypes. **a** For the 39 cultivars, C-type cultivars have lower *OsTb2* expression than the T-type at 40 DAG and 50 DAG under both irrigated and dryland conditions. **b** C-type is associated with fewer tillers under irrigated and dryland conditions. The expression of *OsTb2* is significantly positively correlated with tiller numbers in the 39 cultivars at 50 DAG under dryland (**c**) and irrigated (**d**) conditions. **e** C-type cultivars have significant higher yield per panicle than T-type cultivars in three growth seasons under dryland conditions. **f** The expression of *OsTb2* is negatively correlated with grain yield per panicle in the 39 cultivars in the 3^rd^ season under dryland condition. ‘R’ refers to Pearson product-moment correlation coefficient; ‘*P*’ is *P*-value. Student’s *t*-test analysis indicated a significant difference (*P < 0.05, **P < 0.01). Each dot in a, b and e represents the mean value of about 8 plants and n > 15 cultivars for statistics.

Since SNP3 causes a mutation in the 3’UTR of the *OsTb2* transcript, it is likely that this SNP might alter the expression level of this gene. To determine whether the SNP3 in 3’UTR contribute transcription regulation of *OsTb2*, we grew 39 rice lines with different SNP3 genotypes and checked the expression of *OsTb2* in the tillering node at 40 DAG and 50 DAG (Supplementary Data 2). C-type cultivars consistently showed significantly lower *OsTb2* expression at 50 DAG under both dryland (soil water content, 16.8%) and irrigated conditions (Fig. 6a), suggesting that C-allele of SNP3 reduce the expression of *OsTb2* (Fig. 6a). Consistently, C-type lines produce significantly fewer tillers at both 40 DAG and 50 DAG than T-type lines under both irrigated and dryland conditions (Fig. 6b). Pearson correlation analysis showed that the expression level of *OsTb2* is significantly positively correlated with tiller number at 50 DAG under dryland (Fig. 6c, Student’s *t*-test, *P* = 0.003; R = 0.48) and irrigated (Fig. 6d, Student’s *t*-test, *P* = 0.044; R = 0.35) conditions. Fewer tillers in upland rice represent an adaptive trait because it increases the root/shoot ratio^25,26^ and results in longer deep roots as well as larger panicles^11,12^. To examine whether the C-allele of *OsTb2* selected in upland rice brings about higher grain yield per panicle. We compare the grain yield per panicle data collected in the past three growth seasons between C-type and T-type lines. The results consistently showed that C-type allele is significantly associated with larger yield per panicle in all three growth season under dryland condition (Fig. 6e). We also conducted Pearson Correlation Analysis for the expression of *OsTb2* and yield data (see Methods). We found that *OsTb2* expression is negatively correlated with yield per panicle (Fig. 6f; Student’s *t*-test, *P* = 0.08, R = 0.29), consistent with our findings that the lower *OsTb2* expression confers by the C-allele results in higher yield per panicle under dryland condition. We also tried to examine the correlation between SNP3 and gene expression using our RILs by growing 12 lines in irrigated and dryland conditions in the 2nd season of 2017. However, unfortunately, the dryland came across an extreme drought (soil water content 8.1% at 50 DAG) in that season. As shown in Supplementary Figure 9, we observed the C-allele corresponds to lower gene expression in irrigated conditions, consistent with what we observe in Fig. 6. But unexpectedly, we saw a rapid induction of the C-allele in the extreme drought (Supplementary Fig. 9). We speculated that the extreme drought might have triggered another feedback pathway to compensate the over-suppression of tillering (Supplementary Fig. 9b). The detailed mechanism for this *OsTb2* induction under extreme drought remains to be elucidated by future studies.

## Discussion

Plants modify their development to adapt to the environment, protecting themselves from detrimental conditions by triggering a variety of signalling pathways^27^. Axillary buds are indeterminate structures that can be developmentally controlled in response to endogenous or environmental cues^28^. *OsTb2* from our study is identical to the *RETARDED PALEA1* gene reported in a previous study, which showed that this gene plays a role in palea development and floral zygomorphy in rice^29^. In the present study, we comprehensively analysed the function of *OsTb2* and showed that *OsTb2* modulates the development of axillary buds and was artificially selected during the adaptation of upland rice. Our transgenic experiments and association analysis supported the hypothesis that contrary to *OsTb1*, *OsTb2* suppresses apical dominance and counteracts tillering inhibition by *OsTb1*, leading to an increased tiller number. We found two functional mutations in *OsTb2*, i.e. the 3bp indel-I that distinguishes *japonica* and *indica* subspecies and the SNP3 that differentiates upland *japonica* and irrigated rice (including irrigated *japonica* and *indica*).

In general, *japonica* rice exhibits fewer tillers than *indica* rice, and our data showed that the *japonica-*specific *OsTb2*^*3bp+*^ genotype could increase the number of tillers, while the *indica*-species *OsTb2*^*3bp−*^ genotype could not. This mutation was probably fixed in *japonica* rice because it could alleviate tiller suppression by other genes in the *japonica* background or due to random genetic drift. We’ve shown using transient expression assays that *OsTb2*^*3bp+*^ and *OsTb2*^*3bp−*^ alleles encode proteins with different activities on *D14* expression likely by affecting OsTb2 protein activity (Fig. 4a). Also, using in silico prediction, we showed that this *indel* variant would alter the protein secondary structures of OsTb2, which likely affects its function (Supplementary Fig. 7).

The upland rice ecotype evolved from irrigated *japonica* rice^10^ and adapted to rainfed upland conditions. It has long been well established that in upland rice fewer tillers is an adaptive trait. For example, Fukai et al. screened 1081 rice lines and found that well-adapted upland rice tends to have a small number of well-developed tillers. These lines developed a few large tillers with longer roots^11^, while the lines with profuse tillers tend to have shorter roots and their tillers were not well developed under upland conditions. Also, Kato et al. examined the rice lines adapted to aerobic dryland soils and found that plant architecture with a few large tillers is a more suitable architecture than that with profuse tillers^12^. Consistently, we found that the upland-specific SNP3-C allele has been fixed in upland rice and is associated with tiller reduction in both natural and segregating populations (Tables 1–3). Compared with SNP3-T allele, the SNP3-C allele is associated with lower expression of *OsTb2*^*3bp+*^ under both rainfed upland and irrigated conditions (Fig. 6). Our transgenic data and field experiments based on inbred rice lines both showed that the expression of *OsTb2* positively regulates tiller number, as opposed to the function of *OsTb1*. So, we concluded that the C-type SNP in the 3’-UTR of *OsTb2* is a causal mutation that confers the adaptive fewer tillers in upland rice and was fixed by artificial selection during dryland adaptation.

It should be mentioned that in a growth season in 2017 where our upland field experienced an extreme drought, we observed an unexpected induction of *OsTb2* expression associated with the C-type allele (Supplementary Fig. 9), contrary to what was observed in the irrigated field in that same growth season as well as what we observe in both irrigated and upland fields in this growth season of 2019, we reasoned that it was because the drought stress was so extreme that another feedback pathway might be triggered to compensate the over-suppression of tillering. The detailed mechanism of how C-allele was dramatically induced under extreme drought remains to be elucidated by future studies; this could represent another unknown regulatory pathway. Interestingly, in our association analyses in segregating populations (Tables 2, 3), we repeatedly observed the significant association between SNP3 and tiller number from 40 DAG, but the association between indel-I and tiller numbers only got significant from 50 DAG. The detailed mechanism for this fact is not clear, but given that *OsTb2* has a much higher expression in 40 DAG than in 50 DAG (supplementary Fig. 5), it is very likely that in 40 DAG the effect of gene expression variation caused by SNP3 masks the effect of protein activity difference caused by Indel-I.

So far there have been quite a few genes known to control the tiller formation in rice, such as *MOC1*^30–32^, *SPL* genes^33,34^, *miR156*^35^, *LB4D*^36^, *DWARF10* ^37^, *D14*^16^, *D53*^38^, *RCN8/9*^39^, and *OsTb1*^40^. Some of these genes have strong effects on rice tillering. However, to our knowledge, *OsTb2* is the only gene regulating tiller number in rice determined to have been subjected to artificial selection. The reason that *OsTb2*, rather than other genes, was selected during upland rice adaptation remains elusive. It might have been a chance occurrence. Alternatively, artificial selection may have preferred to act on *Tb1* homologues, given that they have contributed to morphological evolution across different cereal crops. This situation may have arisen because this family of genes can be easily modulated for phenotypic evolution without a considerable detriment to other agronomic traits. This question remains to be further explored. It should be mentioned that, as a regulatory gene, the effects of *OsTb2* on tillering seems to be moderate or even minor in some conditions, because the effect sizes of the two variants (Indel 1 and SNP3) range from 0.47 to 2.77 in the segregating populations (Table 2, Table 3, Supplementary Table 1) and the phenotypes of the transgenic lines are significant but not dramatic (Fig. 3). But selection genes do not have to be large-effect genes. For example, a recent work reported that a minor-effect gene controlling seed dormancy was parallel selected in the domestication of soybean, rice and tomato^41^. The large-effect genes, such as *MOC1*, might cause too severe phenotypes that are not suitable for agricultural production, and consequently are not favored by human selection.

Strigolactone (SLs) signalling and biosynthesis are involved in the regulation of branching in plants^42,43^. The *D14* gene functions in the MAX/RMS/D pathway of SL biosynthesis^16,42^. It was previously reported that OsTb1 regulates tiller development in rice by modulating *D14* expression indirectly^17^; we now report that OsTb2 can interact with OsTb1 and may regulate *D14* expression indirectly by counteracting OsTb1. Consequently, OsTb2 may be involved in balancing the *D14*-mediated SL signalling pathway. Recent studies indicate that TCPs in *Oryza sativa* (rice), *Sorghum bicolor*, and *Arabidopsis thaliana* act downstream of the auxin and MORE AUXILIARY GROWTH (MAX) pathways^44–46,40^. Additional studies are needed to understand whether the regulation of tiller number by *OsTb2* is also associated with auxin pathways.

Evolutionary novelties often originate from gene duplication. In this study, we found that *OsTb2*, as a duplicate gene of *OsTb1*, does not function as a tiller inhibitor but evolved a function opposite that of *OsTb1*, adding an example to the classical concept of neo-functionalization^15,47–50^. There was actually a similar report that detailed the interaction between two isoforms of an important BRANCHED1 (BRC1) transcription factor in potato^51^. In that case, the regular long form inhibits lateral branching, similar to BRC1 in other species, but a modified protein that originates from alternative BRC1 splicing inhibits the long form and promotes lateral branching^51^. In our study, two *Tb1* homologues, *OsTb1* and *OsTb2*, were shown to have antagonistic effects on rice tiller number, similar to the model of the regulation of lateral branching in potato by BRC1 isoforms and the regulation of flowering time in beets that is controlled by the interplay of two paralogs of the *Arabidopsis FLOWERING LOCUS T* (*FT*) gene with antagonistic functions^52^.

While the Mayans had a lucky break discovering plants with the *Tb1* transposon^51^, we are now on the cusp of understanding TCP genes and plant branching. In this context, there is the prospect that regulating *OsTb2* or other *Tb1* homologues will lead to superior outcomes in the adaptation and breeding of rice and other cereal crops.

## Methods

### Plant materials and phenotyping

The 84 upland and 82 irrigated accessions included in this study were collected from different regions worldwide (Supplementary Data 1). One 134 F_8_ recombinant inbred lines (RILs) were generated from F_2_ plants that were obtained by crossing the upland variety IRAT104 and the irrigated variety IR64. We identified RIL116, which was heterozygous for both Indel I and SNP3, and selfed this individual to produce a near-isogenic F_2_ population segregating for both Indel I and SNP3. The derived near-isogenic F_2_ population included 451 individuals, all of which were grown to be phenotyped and genotyped.

Phenotyping was performed in both irrigated and dryland conditions (i.e., preventing soil submergence in water to simulate a rainfed upland environment) for three growth seasons at Xishuangbanna, Yunnan province (1st season refers to the second season of 2015; 2nd season refers to the second season of 2017; 3rd season refers to the first season of 2019). For the irrigated condition, seeds were germinated in a seedbed, and seedlings were then transplanted to a paddy field, where water was ponded on the soil surface throughout the growth and developmental period. For the rainfed upland condition, we conducted direct seeding by dibbling seeds in dry soil. To fully simulate rainfed conditions, no irrigation was applied in the upland condition. When rain came, we drained any excess water to prevent soil submergence. For each accession, we planted three replicates and each replicate have 12 individuals in two rows (6 individuals in each row), with a row spacing of 30 centimetres and a plant spacing of 20 centimetres. For each line, approximately eight individuals were randomly selected and phenotyped. The tiller numbers of the accessions and RILs were surveyed at 40 and 50 DAG, and yield per panicle of the accessions were investigated. The soil water content of dryland was measured by soil moisture meters (TZS-W, Zhejiang Top Instrument Co.Ltd) at 40 and 50 DAG.

### Identification of *OsTb2* using a population genetic approach

*OsTb2* was reported from our previous work^10^. We performed a whole-genome scan for genes with the top *F*_ST_ and XP-CLR signals. We first determined the allele frequencies of the SNP alleles in the upland and irrigated *japonica* populations (Supplementary Data 1) using the resequencing data reported in our previous work^10^. Then based on the allele frequencies, we calculated the *F*_ST_ value between upland and irrigated populations using the method described by Nei^53^. In the genome scan, we used 20-kb sliding windows with 2-kb sliding step. The *F*_ST_ value for each window was obtained by averaging the *F*_ST_ values over SNP sites in that window. To calculate the XP-CLR score, we used the software XP-CLR^54^ and allele frequencies from upland and irrigated populations. A window size of 0.1 cM, a 2-kb grid size and a maximum SNP number of 150 for each window was used. *OsTb2* was found to be located in regions with the top 5‰ *F*_ST_ and XP-CLR signals between upland *japonica* and irrigated *japonica* accessions, which have significantly different tillering abilities^10^. When running the BLAST program against the rice genome using the maize *Tb1* gene sequence, the orthologue *OsTb1* has the highest identity and the paralogue *OsTb2* has the second highest identity. To further substantiate *OsTb2* is a paralogue, we downloaded maize and rice genes in this family from the Panther gene family database and use the MUSCLE software to infer the phylogenetic relationship among these genes (Supplementary Fig. 10)^55^. We also used the MCscan software to do synteny analysis (Supplementary Fig. 11)^56^. Our results supported that *OsTb2* is a paralogue rather than an orthologue of the maize *Tb1* gene.

### Evolutionary analyses detecting *OsTb2* as under selection

For allele frequency spectrum (AFS) analysis, we resequenced the upland and irrigated *japonica* accessions at higher depth of about 15× for more accurate allele frequency estimation. Using the SNP information around the *OsTb2* gene region (from 80 kb downstream to 80 kb upstream) and *indica* rice as outgroup, we generated the derived AFSs for SNPs from windows, which are 10 kb, 20 kb, …, and 80 kb away from the putative causal mutations of the *OsTb2* gene for both upland and irrigated rice populations and then checked if the AFSs display a U-shape pattern, a signal of the hitch-hiking effect. The raw reads that map this gene region can be provided upon request. We further used a nonparametric test (Kolmogorov-Smirnov test) to examine if the U-shape pattern decays with the increasing distance from the focal mutation.

We applied the Hudson-Kreitman-Aguadé (HKA)^57^ and population branch statistic (PBS)^58^ to identify candidate genes having recently reached fixation. Three populations (irrigated *japonica*, upland *japonica* and *indica*) were used to calculate pairwise *F*_ST_ values of SNPs. For all the 44,643 genes, mean *F*_ST_ were generated using SNPs only located in coding regions. Then a classical transformation by Cavalli-Sforza *T*^*pop1−2*^ = −log (1 – *F*ST) was obtained to estimate the divergence time T between Population1 (Pop1) and Population2 (Pop2) in units scaled by population size. The length of population branch can be obtained by Eq. 1:

1 $$PBS_{pop{\it{1}}} = \left( {T^{{\it{pop1 - 2}}} + T^{{\it{pop1 - 3}}} + T^{{\it{pop2 - 3}}}} \right)2^ \wedge - 1$$

Then we recorded the SNPs number (A) of each population and the number (B) of fixed SNPs (the sites with *F*_ST_ > 0.9 for the population compared with both two other populations), performed the HKA test by comparing the ratio of A/B to the genome-wide average and testing the null hypothesis A/B(gene) = A/B(genome-wide) using a Pearson’s Chi-square test on the 2 × 2 contingency table. Finally, genes with PBS value ranking genome-wide top 5% and a significant nominal *P*-value (<0.05) for the HKA test were considered as sweeps candidates. ORF evidence and notes were extracted from rice annotation database.

SweeD^59^ was used for detecting selective sweeps in the upland and irrigated *japonica* populations with the following settings (*−folded −grid 40000*). And the regions with top 5% composite likelihood ratio statistic^60^ were identified as having significant selection signatures.

### Identification of variations around *OsTb2* and genotyping

DNA fragments around the *OsTb2* gene were amplified from the 130 accessions by tb2-up-f/tb2-up-r and tb2-f/tb2-r primers (Supplementary Table 2), and subjected to Sanger sequencing. Seven SNPs and two indels were identified by alignment with MEGA software.

Based on the sequence around SNP3 and Indel I, we designed dCAPS for the genotypes of these loci. For SNP3, a 124 bp fragment was amplified via PCR by tb2-SNP3-f/tb2-SNP3-r primers (Supplementary Table 2) and then cut using the restriction enzyme Bsl I. Two bands (99 bp and 25 bp) were observed in the gel for to the C-genotype, while the T-genotype could not be digested. For Indel I, 200 bp PCR products were obtained with (tb2-indel-gate-f/tb2-indel-r) primers (Supplementary Table 2) and then digested with the restriction enzyme *Bsl* I. Two bands (130 bp and 70 bp) were observed in the gel for to 3bp+ genotype, while 3bp- type could not be cut.

### Association analysis and conditional association analysis

Association analysis was used to test the association between SNP3, Indel I and tiller number. The genotypes of the alleles of 130 accessions were determined using Sanger sequencing, and the genotypes of the RILs of NILs were determined using dCAPS markers (Supplementary Table 2). The accessions were then classified into three different genotypes (two homozygotes and one heterozygote). Student’s *t*-test was subsequently performed to compare the tillers between the two homozygous groups. Conditional analysis: A total of 52 accessions with the T-genotype for the SNP3 site, but different Indel I genotypes were used to test the association between the Indel I genotypes and phenotypes; 50 accessions with the 3bp+ genotype for Indel I, but different genotypes for the SNP3 locus were used to assess the association between SNP3 genotypes and tiller numbers.

### Quantification of gene expression using real-time PCR

We conducted quantitative PCR to survey the expression level of *OsTb2* in different genotypes including 39 cultivars (Supplementary Data 2) and 12 lines from 134 RILs according 3bp+/C and 3bp+/T genotype. Total RNA was extracted from the tiller node tissues of the plant materials at 40 DAG or 50 DAG. After digesting the RNA samples with DNase I (Fermentas), we performed reverse transcription with the Fermentas K1632 Revert Aid H minus First-Strand cDNA kit. We used SYBR-Green Supermix (Bio-Rad) to conduct real-time PCR and analysed the samples in the ABI 7000 Sequence Detection System. β-actin (actin-f/actin-r) was used as an internal control. The OsTb2-specific qPCR primers for the transcript included tb2-qPCR-f and tb2-qPCR-r (Supplementary Table 2).

### Vector construction and genetic transformation

The coding region of *OsTb2* was amplified from rice (IRAT104 and IR64 cultivars, which were the parents of the RIL population used to identify this gene) cDNA by PCR using *Kpn* I and *Bam* H I linker primers (Supplementary Table 2). The resulting *OsTb2* fragment was inserted into the *Kpn* I and *Bam* H I sites of *pCUbi1390*^61^, generating *Ubipro::OsTb2*. All the vectors were introduced into *Agrobacterium tumefaciens* strain *EHA105* and then transferred into Nipponbare plants via *Agrobacterium*-mediated callus transformation^62^. Phenotyping of the T_2_ transgenic lines was performed using the above methods at 25 DAG to 73 DAG.

### Subcellular localization of GFP-OsTb2 fusion proteins

The open reading frames (ORFs) of *OsTb2* were inserted into *pMDC43* as C-terminal fusions with the green fluorescent protein (GFP) reporter gene driven by the CaMV 35 s promoter^63^. These constructs were transformed into the leaves of 3-week-old tobacco (*Nicotiana benthamiana*) by *A. tumefaciens* infiltration^64^. DAPI staining was used to identify the nucleus. The resulting green fluorescence of protoplasts expressing GFP-OsTb2 was observed using a confocal laser-scanning microscope (LSM700, Zeiss, Jena, Germany).

### Bimolecular fluorescence complementation assay

Complementary DNAs of *OsTb2* and *OsTb1* were cloned into the bimolecular fluorescence complementation (BiFC) vectors *pnYFP-X* and *pcCFP-X*, respectively, with GUS also cloned as a negative control. The constructs were cotransformed into *Nicotiana benthamiana* protoplasts for transient expression. Protoplast isolation from tobacco leaf tissues and PEG-mediated transformation were performed according to Bart et al.^65^. Cells were incubated at 28 °C in the dark overnight. A confocal laser-scanning microscope (LSM700, Zeiss, Jena, Germany) was used to observe the green fluorescence of protoplasts. The *35S::GFP* construct and AHL22 were used as a control and a nuclear marker protein, respectively^66^.

### Protein coimmunoprecipitation assay

The recombinant constructs *GFP-OsTb2* and *MYC-OsTb1* were introduced into rice protoplasts, and protein extracts were prepared as described by He^67^. The protein extracts were precipitated with anti-GFP agarose beads (CMC Scientific, http://www.cmcscientific.com) overnight. Then, proteins bound to the beads were resolved by SDS-PAGE and detected by Western blotting using anti-GFP (dilution at 1:1000; ab1218, abcam), anti-MYC (dilution at 1:1000; ab264433, abcam) primary antibodies (MBL, http://www.mblintl.com/) and HRP-labelled goat anti-mouse secondary antibody (dilution at 1:5000; ab97023, abcam).

### Transient expression assays in rice protoplasts

For the *D14* promoter repression assay, two forms of *OsTb2* were used in the system. The full-length *OsTb2* cDNAs were fused into the *pRTVcMyc* vector, driven by the 35 s promoter, to generate *pRTVcMyc-OsTb2*^*3bp+*^ and *pRTVcMyc-OsTb2*^*3bp−*^. To generate the *D14pro::LUC* reporter gene, the *D14* promoter (*D14pro*) was amplified. The plasmid carrying the GUS gene under the control of the 35 s promoter was used as a normalization control. The presented values represent the means ± s.d. of six technical replicates. Cotransformation of the *D14pro::LUC* reporter and *pRTVcMyc-OsTb2*^*3bp+*^ or *pRTVcMyc-OsTb2*^*3bp−*^ was performed according to He et al.^67^ to identify the effect of OsTb2 in the transient assay. The *Renilla* luciferase reporter gene (*REN*) under the control of CaMV35S promoter was used as an internal control to normalize the data for eliminating variations in the experiment.

### Dual-luciferase assays in tobacco leaves

The effector plasmids *pMDC43-OsTb2*, *pMDC43-OsTb1*, and *pMDC43-OsMADS57* were cloned as described above. The reporter plasmid pGreen-D14pro-LUC encodes two luciferases, firefly luciferase controlled by the *D14* promoter and the *Renilla* luciferase controlled by the constitutive 35 s promoter. The *D14pro*, fused to the minimum 35 s promoter, was PCR amplified from the 35 s template and cloned into the Hind III/Bam HI sites of the vector *pGreen-0800-LUC*. *pGreen-D14pro-LUC* was transformed into Agrobacterium (strain *EHA105*) carrying the helper plasmid *pSoup-P19*, which also encodes a repressor of co-suppression^68^. The Agrobacterium strain containing both the reporter *pGreen-D14pro-LUC* and the helper *pSoup-P19* was used either alone or mixed with the Agrobacterium strain containing the effector plasmids *pMDC43-OsTb2*, *pMDC43-OsTb1*, and *pMDC43-OsMADS57*, as shown in Fig. 4b. *pMDC43-OsGRF4* was used as a negative control effector. Overnight cultures of Agrobacterium were collected by centrifugation resuspended, and infiltrated as described above^62^. After 3 days, using commercial Dual-LUC reaction (DLR) reagents according to the manufacturer’s instructions (Promega) leaf samples were collected for the Dual-LUC assay. Specifically, we excised leaf discs from the site (ca. 1-2 cm in diameter) of Agrobacterium infection, ground using liquid nitrogen, and homogenized using 100 μl of Passive Lysis buffer (Promega). Then, we mixed 20 μl of the crude extract with 100 μl of Luciferase Assay buffer (Promega), and examined the firefly luciferase activity (LUC) using a luminometer (BG-1, GEM Biomedical Inc). After the measurement of firefly luciferase activity, 100 μl of Stop and Glow buffer (Promega) was added to quench the firefly luciferase and initiate the *Renilla* luciferase reaction.

### Yeast one-hybrid assay using the pLacZi2u/pB42AD system

We followed the previous reported procedure^69^. Briefly, the coding sequence of *OsTb2* was inserted into the MCS of *pB42AD* to generate an AD-fusion construct (*pB42AD-OsTb2*), and *D14pro* was inserted into the MCS of the pLacZi2μ reporter plasmid (*pLacZi2μ-D14pro*). The *pB42AD-OsTb2* plasmid was cotransformed with *pLacZi2μ-D14pro*, including the *LacZ* reporter gene driven by a *D14pro* fragment, for testing in *EGY48* yeast strain. Transformants were grown on SD (galactose+raffinose)/-Ura/-Trp/X-gal plates.

### Reporting summary

Further information on research design is available in the Nature Research Reporting Summary linked to this article.

## Supplementary information

Supplementary information

Reporting Summary

Description of Additional Supplementary Files

Supplementary Data 1-2

## Data Availability

Data supporting the findings of this work are available within the paper and its Supplementary Information files. A reporting summary for this Article is available as a Supplementary Information file. The datasets generated and analyzed during the current study are available from the corresponding author upon request. DNA-seq data were deposited in in the National Center for Biotechnology Information (NCBI) under the SRA accession number PRJNA595072. The source data underlying Fig. 5b as well as Tables 1, 2, and 3 are provided as a Source Data file.

## Source data

Source Data
