# Supplementary material for: Neo-functionalization of a Teosinte branched 1 homologue mediates adaptations of upland rice
Source: Nat Commun. 2020 Feb 5;11:725. doi: 10.1038/s41467-019-14264-1 (PMC7002408; doi:10.1038/s41467-019-14264-1)
Supplement: Supplementary file 1 — Supplementary information [file 41467_2019_14264_MOESM1_ESM.pdf]

Neo-functionalization of a *Teosinte branched 1* homologue mediates adaptations of upland rice

Lyu *et al.*

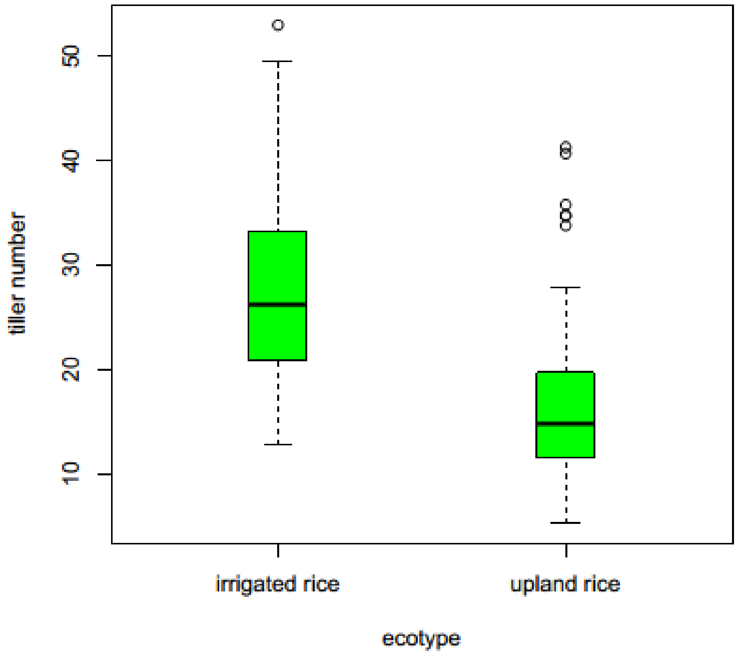

Supplementary Fig. 1. Tiller numbers of irrigated and upland rice under both irrigated and dryland conditions. n = 8 plants per variety; 3 replicates.

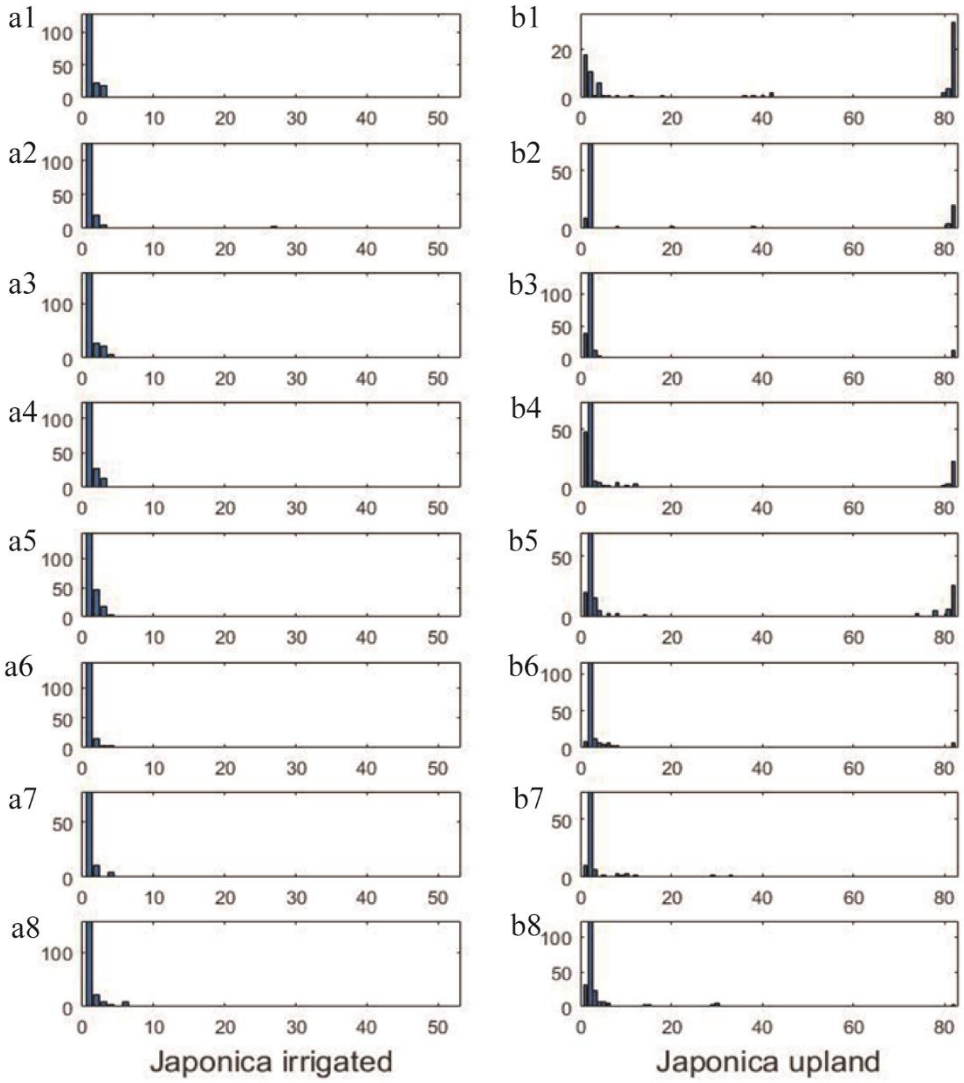

Supplementary Fig. 2. The derived allele frequency spectra of SNPs from windows with a size of 20 kb near the causal mutation. Subplots (**a1**) and (**b1**) are allele frequency spectra (AFS) for SNPs from the region that spans 10 kb to the left of the causal mutation and 10 kb to the right of the causal mutation (e.g., [-10 kb, 0] & [0, 10 kb]); Subplots (**a2**) and (**b2**) are AFS for SNPs from the [-20kb, -10kb] & [10kb, 20kb] regions; And similarly the other subplots were constructed in the same manner.

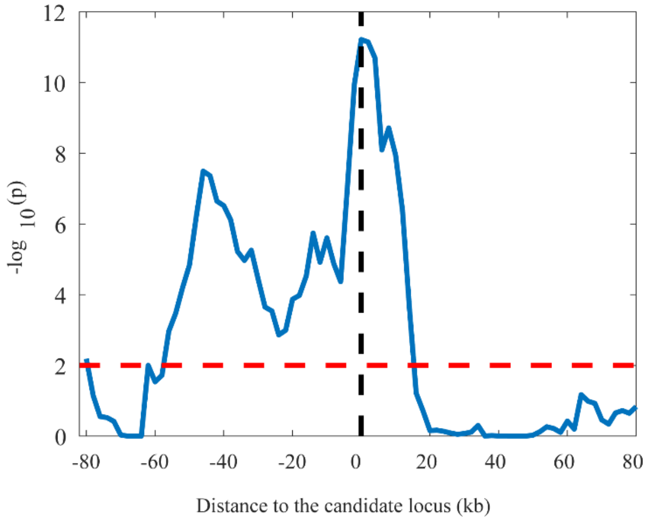

Supplementary Fig. 3. Kolmogorov-Smirnov test of the AFS along continuous windows around *OsTb2* in upland and irrigated *japonica*. The red dashed line corresponds to Kolmogorov-Smirnov test *P* value 0.01. We used window size of 20 kb and step size of 2 kb. The horizontal ordinate represents distance of the center of window to the candidate locus on Chromosome 9.

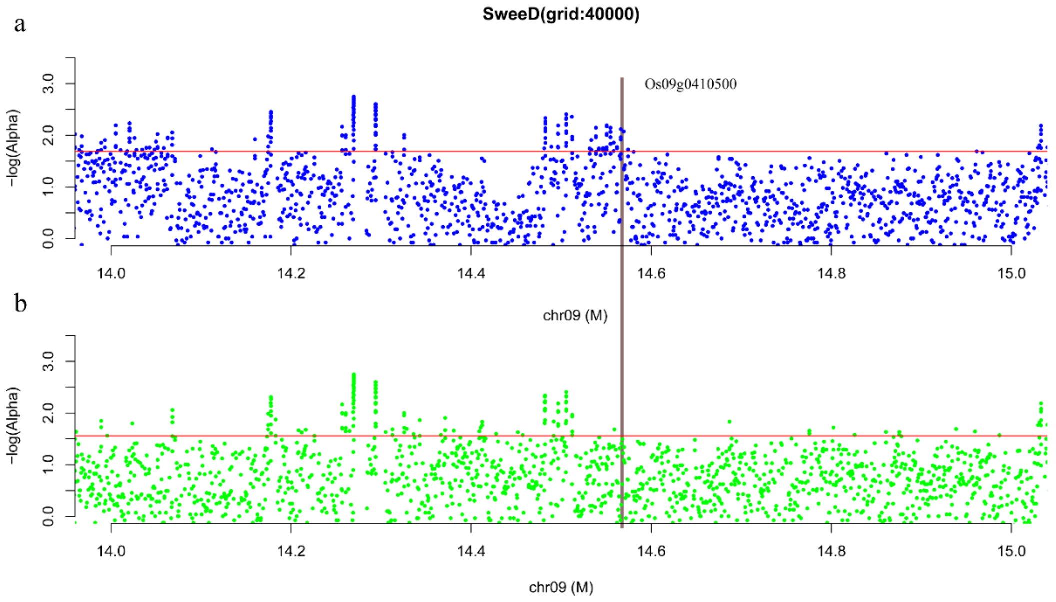

Supplementary Fig. 4. The detection of selection signals using SweeD. The figure demonstrates the signals identified in the region spanning 1Mb on chromosome 09 including *OsTb2*. The top panel (**a**) shows the alpha values in upland *japonica*, while the bottom panel (**b**) shows the alpha values in irrigated *japonica*. The red line shows the threshold value for top 5% windows. The vertical gray line showed the position of *OsTb2*. It can be seen that around the region containing *OsTb2*, the signal is very significant in upland rice, but not in irrigated rice.

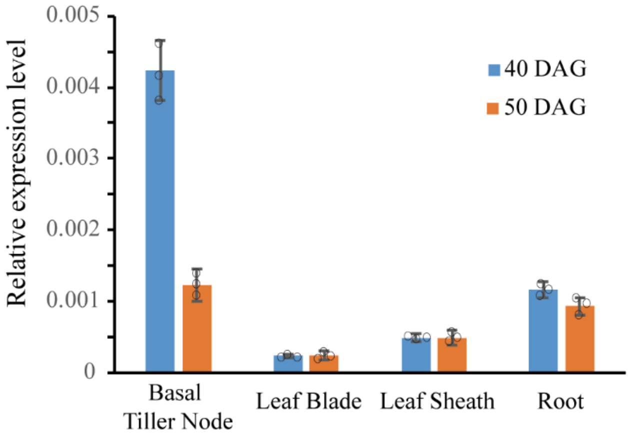

Supplementary Fig. 5. Expression model of *OsTb2* in different tissues of rice at 40 DAG and 50 DAG under irrigated condition. A typical upland rice cultivar IRAT104 was used as material.

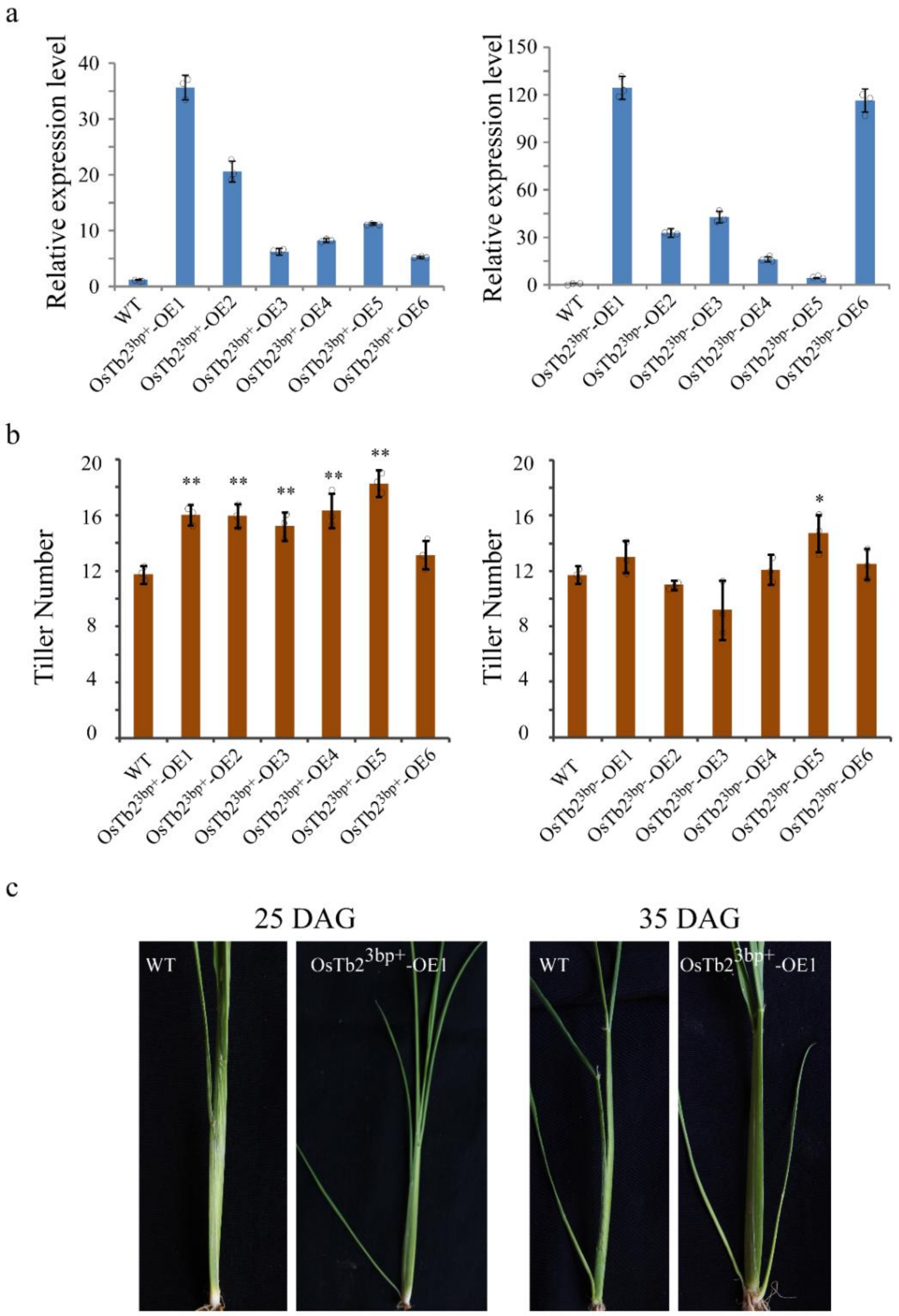

Supplementary Fig. 6. Phenotypic analysis of transgenic plants overexpressing *OsTb2*$^{3bp+}$ and *OsTb2*$^{3bp-}$. a Expression analysis of *OsTb2* in transgenic plants overexpressing *OsTb2*$^{3bp+}$ and *OsTb2*$^{3bp-}$, respectively. **b** Tiller numbers of transgenic plants overexpressing *OsTb2*$^{3bp+}$ and *OsTb2*$^{3bp-}$. Student's *t*-test analysis indicated a significant difference when compared with WT (*$P < 0.05$, **$P < 0.01$). Each value represents the mean ± s.d. (n = 24 plants; 3 replicates). **c** Morphology of the shoot apical meristem and axillary meristem determined by stripping leaves from WT and OsTb2^{3bp+}-overexpressing transgenic plants at 25 DAG and 35 DAG.

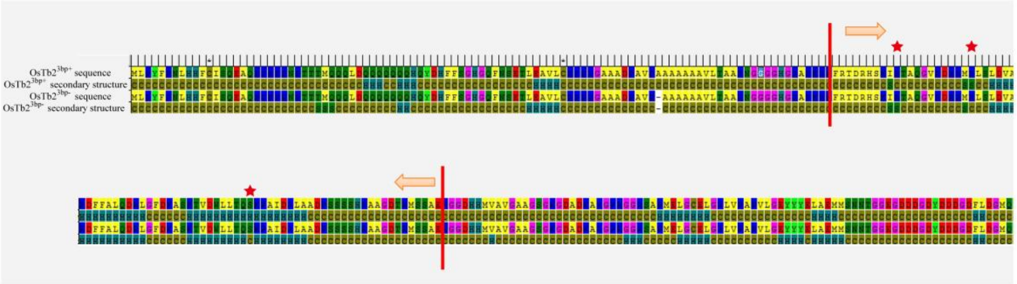

Supplementary Fig. 7. Comparison of secondary structures of OsTb2^{3bp+} and OsTb2^{3bp-} proteins. C indicates a random coil; H indicates an α-helix; S indicates a β-pleated sheet. The 104~184 amino acids flanked by red vertical lines constitute the TCP binding domain. The red stars indicate potential secondary structure differences between the two isoforms.

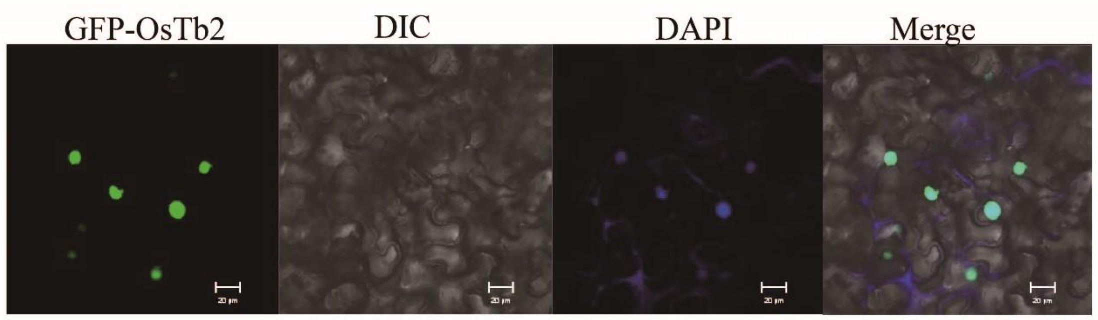

Supplementary Fig. 8. Confocal scanning images showing nuclear localization of the GFP-OsTb2 fusion protein in *Nicotiana benthamiana* leaves following *A. tumefaciens* infiltration. GFP-OsTb2 indicates fluorescence signal under blue laser, demonstrating that OsTb2 is located in the nucleus of plant cell. DIC indicates differential interference contrast transmission. DAPI indicates nucleus staining, which can be seen as nucleus maker. The merged image is shown on the right. Scale bars, 20 μm.

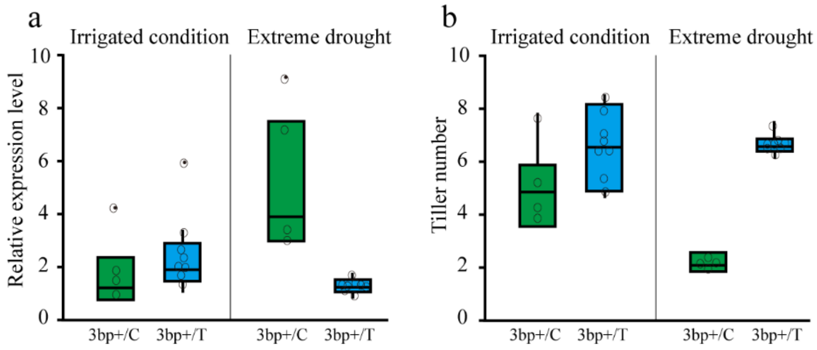

Supplementary Fig. 9. Assessing the effects of SNP3 on *OsTb2* expression and tiller numbers using 12 RILs under an irrigated condition and an extreme drought condition. Extreme drought condition: soil water content 8.1%. **a** Under irrigated condition, the *OsTb2* expression was not significantly different between C-type and T-type lines (note the trend is that C corresponds to lower expression), while under extreme drought the *OsTb2* expression is dramatically up-regulated. **b** Tiller numbers of T- and C-type RILs with *OsTb2*$^{3bp+}$ genotype under both irrigated and extreme drought conditions. Each dot in a and e represents the mean value of about 8 plants and n = 4 in 3bp+/T and 8 in 3bp+/C RIL for statistics.

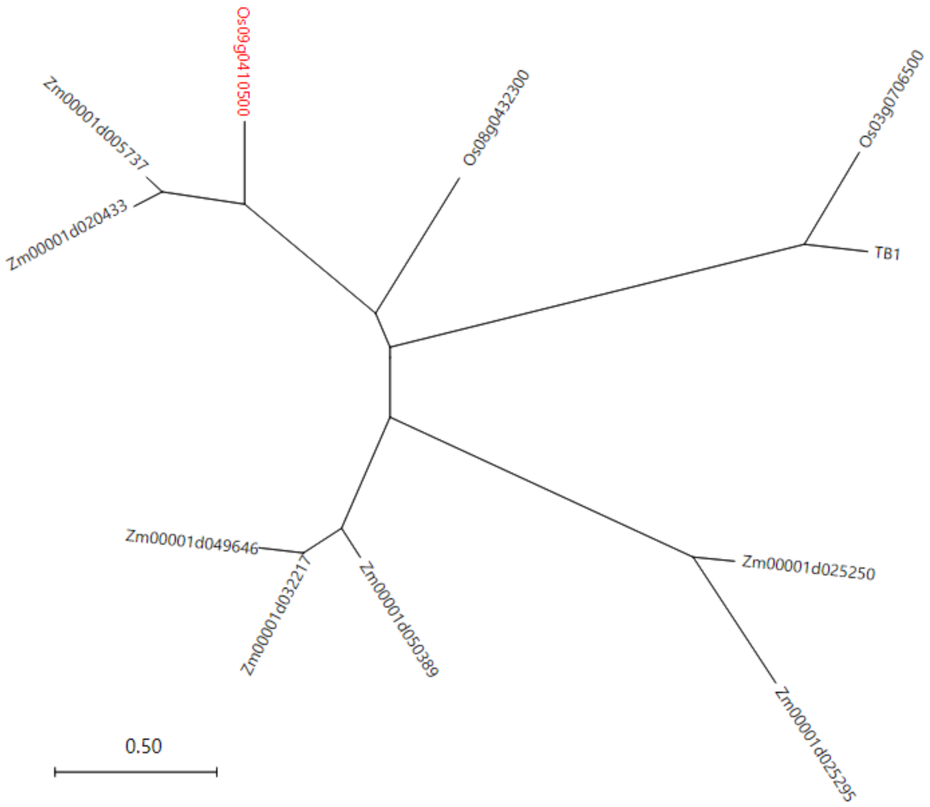

Supplementary Fig. 10. Phylogenetic relationship of Tb1 and closely related genes in rice and maize. Genes were selected from Panther gene family database and their sequence were downloaded from NCBI. Protein sequences were aligned using MUSCLE and protein sequence distance trees were inferred using Maximum Likelihood method and JTT matrix-based model. Gene identifiers of rice and maize start with 'Os' and 'Zm', respectively, and Os09g0410500 (OsTb2) is shown in red. OsTb2 is the ortholog of Zm00001d020433 and Zm00001d005737, but not Tb1 of maize.

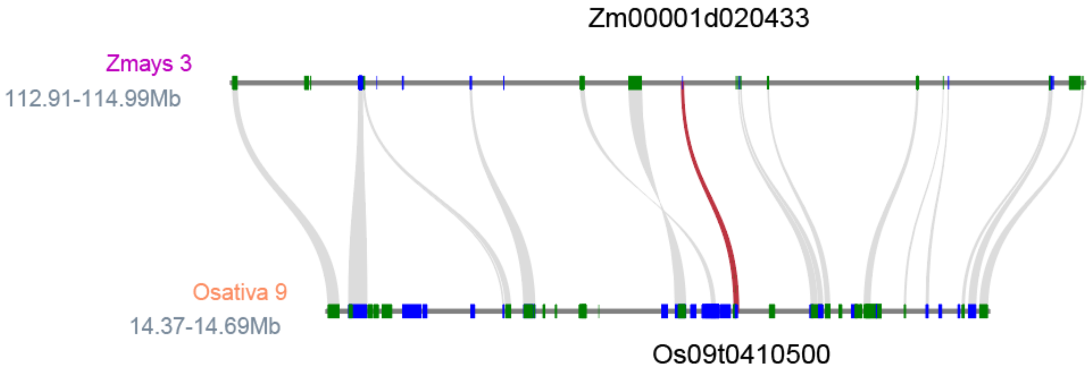

Supplementary Fig. 11. The Synteny block between rice and maize containing *OsTb2*. Os09g0410500: *OsTb2*. Synteny blocks between rice and maize were calculated using MCscan. The upper block is maize (112.91-114.99 Mb on chromosome 3) and the lower is rice (14.37-14.69 Mb on chromosome 9). The dark red ribbon connects *Os09g0410500* and *Zm00001d020433*, which are orthologs of each other.

Supplementary Table 1. Tiller number analysis of each genotype in natural accessions.

Genotype	40 DAG					50 DAG				
	Median Tiller Number	Mean Tiller Number	Variance	Standard Deviation	Effect Size	Median Tiller Number	Mean Tiller Number	Variance	Standard Deviation	Effect Size
Indel1-3bp+	5	6.3	8.7	2.9	0.8	17.2	17.6	95.9	9.8	7
Indel1-3bp-	8	7.9				31.8	31.6			
SNP3 - C	5	5.4	8.8	3	1.2	14.7	14.9	102.1	10.1	6.15
SNP3 - T	8	7.8				26.3	27.2			

Supplementary Table 2. Primers used in this study.

Name of primer	sequence (5'-3')	Purpose
tb2-up-f	TCCCCAAATAGCCATATTCCC	Genotyping
tb2-up-r	TGTGCATCGAAGCTTAAGGA	
tb2-f	CAAACAAACCCTTGTGGTCAG	Genotyping
tb2-r	TAGACAGATAAGCAAAAGCGAGA	
tb2-SNP3-f	GCAGATCAAGACCTAATCCCTCAATC	Genotyping for SNP3
tb2-SNP3-r	GACGGTATGCAATATTAGCTTCAG	
tb2-indel-gate-f	GGACAAGTTTGTACAAAGCAGGCTGGGACCACTTTGTACAAGAAAG	Genotyping for Indel 1
tb2-indel-r	ACAGCCGCATCCGGCGGTCGCGC	
actin-f	TCAACCCAAGGCCAATC	qPCR
actin-r	CACCATCACCAGAGTCCAACA	
tb2-qPCR-f	AACAACACCGGAGGAGAAG	qPCR
tb2-qPCR-r	TAGCAGATCAAGACCTAATCAATC	
Tb2-Kpn1-cds-F	AGTCggtaccATGTTGCCTTACTTTCCTAACCT	Clone for pCUbi1390
Tb2-BamH1-cds-R	TGACggatccCTAATATTGCATACCGTCCAAG	
Tb2-cds-F	ATGTTGCCTTACTTTCCTAACCT	Clone for pGWC, a gateway cloning vector with attL1 and attL2
Tb2-cds-R	CTAATATTGCATACCGTCCAAG	
Tb1-cds-F	ATGCTTCCTTTCTTCGATTCCC	Clone for pGWC, a gateway cloning vector with attL1 and attL2
Tb1-cds-R	TCAGCAGTAGTGCCGCGAAT	
MADS57-cds-F	ATGGGGAGGGGGAAGATAGT	Clone for pGWC, a gateway cloning vector with attL1 and attL2
MADS57-cds-R	TTAAGGCAGATGAAGTCCCAG	

D14Pro-F1	TCGACGGTATCGATAAGCTTAGCAGGAAATCCATCAGGACCCG	Clone for pGreen0800
D14Pro-R1	CTAGTGGATC CCCCGGGC CACACCAGCGCGGCGGATT	
Tb2-Kpn1-cds-F	AGTCggtaccATGTTGCCTTACTTTCCTAACCT	Clone for pRTVcMyc
Tb2-Spe1-cds-R	TGACactagtCTAATATTGCATACCGTCCAAG	
D14Pro-F2	ATCTGTCGACCTCGAGGAGCCACCACAGAAGATAAAG	Clone for pLacZi2μ
D14Pro-R2	GAGCACATGCCTCGAGCTTCAGTTATTTAGGGGGGTG	
Tb2-IF-F	TGCCTCTCCCGAATTCATGTTGCCTTACTTTCCTAACCT	Clone for pB42AD
Tb2-IF-R	CGAGTCGGCCGAATTCCTAATATTGCATACCGTCCAAG	
