# Supplementary material for: Neo-functionalization of a Teosinte branched 1 homologue mediates adaptations of upland rice
Source: Nat Commun. 2020 Feb 5;11:725. doi: 10.1038/s41467-019-14264-1 (PMC7002408; doi:10.1038/s41467-019-14264-1)
Supplement: Supplementary file 3 — Description of Additional Supplementary Files [file 41467_2019_14264_MOESM3_ESM.docx]

**Description of Additional Supplementary Files**

File name: Supplementary Data 1
Description: Basic information of the 84 upland and 82 irrigated accessions sequenced for the OsTb2 locus using Sanger sequencing

File name: Supplementary Data 2
Description: OsTb2 expression and phenotype analysis of 39 selected accessions
