# Supplementary material for: Neo-functionalization of a Teosinte branched 1 homologue mediates adaptations of upland rice
Source: Nat Commun. 2020 Feb 5;11:725. doi: 10.1038/s41467-019-14264-1 (PMC7002408; doi:10.1038/s41467-019-14264-1)
Supplement: Supplementary file 4 — Supplementary Data 1-2 [file 41467_2019_14264_MOESM4_ESM.zip › data 2.docx]

**Supplementary** **Data 2** *OsTb2* expression and phenotype analysis of 39 selected accessions.

| Sample No. | Accession name | Irrigated condition | | | | Dryland condition | | | | Dryland condition | | | | SNP3-  type |
| --- | --- | --- | --- | --- | --- | --- | --- | --- | --- | --- | --- | --- | --- | --- |
|  |  | 40 DAG | | 50 DAG | | 40 DAG | | 50 DAG | | 1st season | | 2nd season | 3rd season |  |
|  |  | Relative expressionl of OsTb2 (2^-△Ct^) | Tiller number | Relative expressionl of OsTb2 (2^-△Ct^) | Tiller number | Relative expression of OsTb2 (2^-△Ct^) | Tiller number | Relative expression of OsTb2 (2^-△Ct^) | Tiller number | Yield per panicle (g) | | | |  |
| GS108 | Boyegu | 0.03316 | 1.1 | 0.00089 | 1.2 | 0.00019 | 3.9 | 0.00303 | 3.8 | 1.06 | 1.87 | | 4.05 | C |
| GS117 | Huangkehongmangjingzhan | 0.00187 | 1.1 | 0.00061 | 1.3 | 0.00035 | 3.5 | 0.00241 | 3.5 | -- | 1.43 | | 1.83 | C |
| GS107 | Sanlicun | 0.00410 | 1.0 | 0.00079 | 1.4 | 0.00032 | 2.4 | 0.00224 | 3.5 | 1.07 | 1.01 | | 2.94 | C |
| GS083 | Xiaobaigu | 0.00770 | 1.0 | 0.00037 | 1.4 | 0.00022 | 4.2 | 0.00248 | 3.9 | 1.23 | -- | | 3.34 | C |
| GS116 | Shanekuai | 0.07126 | 1.0 | 0.00104 | 1.4 | -- | -- | 0.00305 | 2.5 | 2.53 | 1.93 | | 5.43 | C |
| GS093 | Huangpigu | 0.00181 | 1.1 | 0.00056 | 1.5 | 0.00008 | 4.2 | 0.00171 | 3.7 | 1.85 | 0.69 | | 3.31 | C |
| GS143 | IRAT 104 | 0.00423 | 1.1 | 0.00049 | 1.6 | 0.00024 | 4.7 | 0.00116 | 4.7 | 3.18 | 1.50 | | 3.82 | C |
| GS150 | CICA 9 | 0.00450 | 1.0 | 0.00019 | 1.7 | 0.00018 | 3.3 | 0.00336 | 3.0 | 1.30 | 1.26 | | 2.65 | C |
| GS090 | Bayuenuo | 0.00176 | 1.1 | 0.00044 | 1.7 | 0.00010 | 4.8 | 0.00294 | 4.8 | 0.69 | 0.60 | | 1.90 | C |
| GS140 | TGR 78 | 0.00306 | 1.0 | 0.00030 | 1.8 | 0.00013 | 4.0 | 0.00173 | 3.6 | 1.77 | 1.60 | | 3.90 | C |
| GS141 | CIRAD 391 | 0.00555 | 2.3 |  |  | 0.00014 | 3.3 | 0.00092 | 2.9 | 1.10 | 1.25 | | 2.45 | C |
| GS135 | WAB56-125 | 0.00121 | 1.1 | 0.00127 | 2.1 | 0.00034 | 3.4 | 0.00197 | 3.0 | 2.60 | 2.37 | | 3.55 | C |
| GS131 | IAC 25 | 0.00226 | 1.0 | 0.00282 | 2.1 | 0.00026 | 3.2 | 0.00127 | 3.2 | 1.82 | 1.67 | | 3.80 | C |
| GS138 | GUARANI | 0.00295 | 1.6 | -- | -- | -- | -- | 0.00093 | 3.3 | 1.70 | 1.90 | | 2.02 | C |
| GS096 | Mazigu | -- | -- | 0.00094 | 2.1 | 0.00014 | 3.1 | 0.00151 | 3.3 | 0.41 | 0.52 | | 2.33 | C |
| GS097 | Hongzaogu | 0.01645 | 1.3 | 0.00076 | 2.0 | -- | -- | 0.00215 | 3.3 | 1.05 | 0.67 | | 2.35 | C |
| GS139 | Dourado | -- | -- | -- | -- | 0.00038 | 3.5 | 0.00169 | 3.4 | -- | 2.27 | | 4.37 | C |
| GS137 | CNA 4140 | 0.01512 | 1.7 | 0.00101 | 3.2 | 0.00043 | 3.4 | 0.00226 | 3.4 | 2.10 | 1.46 | | 2.15 | C |
| GS147 | AZUCENA | 0.02024 | 1.1 | 0.00059 | 1.8 | 0.00226 | 3.2 | -- | -- | 3.75 | 1.13 | | 5.22 | C |
| GS109 | Liandaogu | -- | -- | 0.00249 | 1.8 | 0.00023 | 3.7 | 0.00063 | 4.2 | 0.76 | 0.85 | | 3.18 | C |
| GS019 | Liao 942 | 0.15141 | 1.6 | -- | -- | 0.00213 | 2.9 | 0.00270 | 2.5 | 0.23 | 0.63 | | 2.37 | T |
| GS020 | Shennong 8712 | -- | -- | -- | -- | 0.00029 | 2.8 | 0.00326 | 3.3 | 0.30 | 0.97 | | 2.79 | T |
| GS002 | IR 29 | 0.05555 | 3.3 | 0.00128 | 7.6 | 0.00052 | 7.1 | 0.00295 | 7.7 | 1.12 | 0.53 | | 2.09 | T |
| GS036 | RD 23 | 0.00559 | 3.4 | 0.00088 | 7.5 | -- | -- | 0.00135 | 7.7 | 1.02 | 0.41 | | 2.76 | T |
| GS161 | B6136-3-TB-0-1-5 | 0.00578 | 3.3 | 0.00106 | 6.1 | 0.00098 | 6.4 | 0.00117 | 7.7 | 1.66 | 0.85 | | 2.39 | T |
| GS015 | Jinmazhan | -- | -- | -- | -- | 0.00083 | 6.7 | 0.00222 | 8.6 | 0.92 | 0.62 | | 3.07 | T |
| GS036 | RD 23 | 0.00498 | 3.3 | 0.00152 | 7.6 | 0.00032 | 6.5 | 0.00198 | 9.4 | 0.66 | 1.76 | | 2.45 | T |
| GS005 | IR 43 | 0.05450 | 3.5 | 0.00086 | 7.7 | 0.00031 | 6.1 | 0.00269 | 6.9 | 2.09 | 0.96 | | 3.84 | T |
| GS075 | IR 20 | 0.00273 | 4.0 | 0.00139 | 8.3 | -- | -- | 0.00104 | 6.6 | 0.56 | 0.87 | | 3.24 | T |
| GS077 | IR 28 | 0.00276 | 3.5 | 0.00467 | 8.5 | 0.00054 | 6.4 | -- | -- | 1.16 | 0.67 | | 1.24 | T |
| GS007 | IR 56 | 0.01881 | 3.6 | 0.00134 | 8.6 | 0.00026 | 9.2 | 0.00524 | 10.0 | 1.00 | 0.95 | | 2.04 | T |
| GS021 | Miyang 46 | -- | -- | 0.00060 | 8.9 | -- | -- | 0.00404 | 9.7 | 0.42 | 1.13 | | 2.84 | T |
| GS006 | IR 50 | 0.02246 | 3.2 | 0.00098 | 9.0 | 0.00020 | 7.1 | 0.00661 | 8.6 | 0.36 | 0.80 | | 1.77 | T |
| GS074 | Dianrui 449 | -- | -- | 0.00306 | 9.1 | -- | -- | -- | -- | -- | 1.43 | | 1.48 | T |
| GS003 | IR 30 | 0.02672 | 4.2 | 0.00238 | 10.9 | 0.00045 | 6.2 | 0.00214 | 7.6 | 0.87 | 1.10 | | 1.85 | T |
| GS009 | IR 74 | 0.02813 | 4.4 | 0.00084 | 11.3 | 0.00016 | 7.5 | 0.00375 | 8.7 | 1.27 | 0.73 | | 1.83 | T |
| GS004 | IR 36 | 0.02006 | 5.4 | 0.00113 | 14.8 | 0.00023 | 9.1 | 0.00699 | 10.9 | 0.70 | 1.23 | | 2.25 | T |
| GS003 | IR 30 | 0.02672 | 4.2 | 0.00238 | 10.9 | 0.00045 | 6.2 | 0.00214 | 7.6 | 0.87 | 1.10 | | 1.85 | T |
| GS157 | B3619C-7B-8-1-4 | -- | -- | 0.00246 | 4.0 | -- | -- | 0.00114 | 7.6 | 0.40 | 1.92 | | 4.19 | T |
