# Supplementary material for: Neo-functionalization of a Teosinte branched 1 homologue mediates adaptations of upland rice
Source: Nat Commun. 2020 Feb 5;11:725. doi: 10.1038/s41467-019-14264-1 (PMC7002408; doi:10.1038/s41467-019-14264-1)
Supplement: Supplementary file 5 — Source Data [file 41467_2019_14264_MOESM5_ESM.zip › source data for fig5b.pptx]

## Slide 1
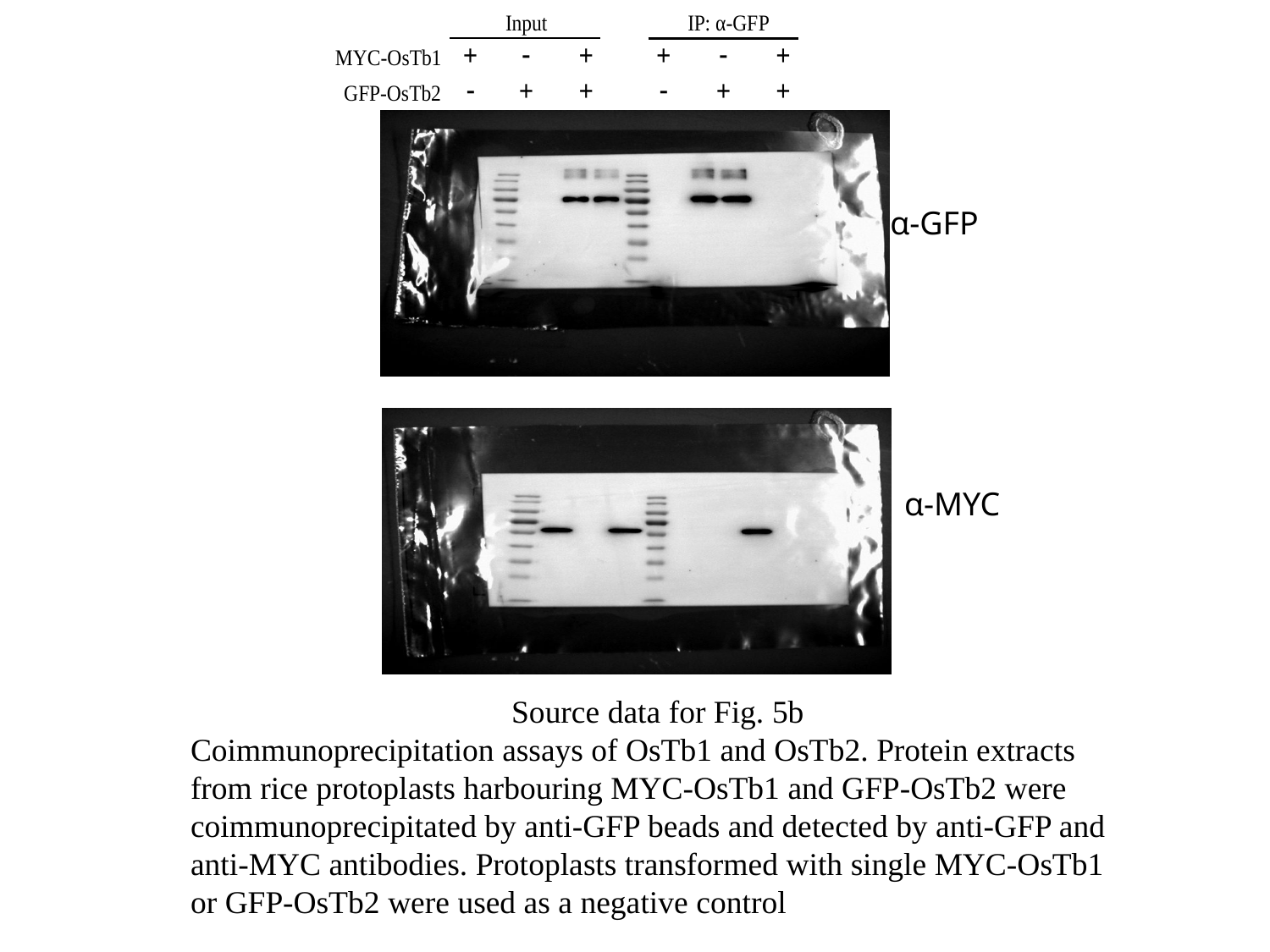

α-GFP
α-MYC
Source data for Fig. 5b
Coimmunoprecipitation assays of OsTb1 and OsTb2. Protein extracts from rice protoplasts harbouring MYC-OsTb1 and GFP-OsTb2 were coimmunoprecipitated by anti-GFP beads and detected by anti-GFP and anti-MYC antibodies. Protoplasts transformed with single MYC-OsTb1 or GFP-OsTb2 were used as a negative control
